# New Insights on the Activity and Selectivity of MAO-B Inhibitors through In Silico Methods

**DOI:** 10.3390/ijms24119583

**Published:** 2023-05-31

**Authors:** Liliana Pacureanu, Alina Bora, Luminita Crisan

**Affiliations:** “Coriolan Dragulescu” Institute of Chemistry, 24 Mihai Viteazu Ave., 300223 Timisoara, Romania; pacureanu@acad-icht.tm.edu.ro (L.P.); alina_bora@acad-icht.tm.edu.ro (A.B.)

**Keywords:** monoamine oxidase B, selectivity, activity cliff, ECFP4

## Abstract

To facilitate the identification of novel MAO-B inhibitors, we elaborated a consolidated computational approach, including a pharmacophoric atom-based 3D quantitative structure–activity relationship (QSAR) model, activity cliffs, fingerprint, and molecular docking analysis on a dataset of 126 molecules. An AAHR.2 hypothesis with two hydrogen bond acceptors (A), one hydrophobic (H), and one aromatic ring (R) supplied a statistically significant 3D QSAR model reflected by the parameters: R^2^ = 0.900 (training set); Q^2^ = 0.774 and Pearson’s R = 0.884 (test set), stability s = 0.736. Hydrophobic and electron-withdrawing fields portrayed the relationships between structural characteristics and inhibitory activity. The quinolin-2-one scaffold has a key role in selectivity towards MAO-B with an AUC of 0.962, as retrieved by ECFP4 analysis. Two activity cliffs showing meaningful potency variation in the MAO-B chemical space were observed. The docking study revealed interactions with crucial residues TYR:435, TYR:326, CYS:172, and GLN:206 responsible for MAO-B activity. Molecular docking is in consensus with and complementary to pharmacophoric 3D QSAR, ECFP4, and MM-GBSA analysis. The computational scenario provided here will assist chemists in quickly designing and predicting new potent and selective candidates as MAO-B inhibitors for MAO-B-driven diseases. This approach can also be used to identify MAO-B inhibitors from other libraries or screen top molecules for other targets involved in suitable diseases.

## 1. Introduction

The monoamine oxidase A (MAO-A) and monoamine oxidase B (MAO-B) are mitochondrial-bound isoenzymes that are responsible for the oxidative deamination of monoamine neurotransmitters, including adrenaline, noradrenaline, serotonin, norepinephrine, β-phenylethylamine, dopamine (DA), dietary amines, e.g., tyramine, etc. [1]. The two isozymes display particular substrate and inhibitor specificities: MAO-A shows better compatibility for hydroxylated amines noradrenaline and serotonin, while MAO-B interacts with non-hydroxylated amines such as benzylamine and beta-phenylethylamine. However, DA and tyramine have a similar affinity for each isoform [1,2,3]. Because of the neurotoxic potential of MAO’s byproducts, including hydrogen peroxide, ammonia, various aldehydes, etc., overactivity of these isozymes can induce mitochondrial damage, depression, Alzheimer’s disease, Parkinson’s disease, etc. [1,2]. The deficiency of dopamine, noradrenaline, and serotonin represents the biochemical substratum of fundamental degenerative mechanisms of Parkinson’s disease (PD) [3]. In keeping with this hypothesis, the development of small chemical entities with inhibitory potential against MAOs generated significant breakthroughs in the therapy of various neuropsychiatric diseases, e.g., neurodegenerative, PD, etc. Starting from the 60s, many MAO inhibitors were developed by researchers, among which the most therapeutically essential drugs are phenelzine, tranylcypromine, pargyline, selegiline, clorgyline, moclobemide, rasagiline, safinamide, ladostigil, etc. The beneficial effect of selegiline in PD patients triggered intensive MAO inhibitors drug development programs for neuropsychiatric disorders [3]. The development of selective irreversible inhibitors for MAOs contributed to the partial elucidation of problematic issues related to the clinical administration of MAO inhibitors [4,5]. In addition to health improvement and in addition to psychiatric and neurological disorders, off-target effects, safety issues, and dietary restraints, i.e., low tyramine intake, accompany the administration of MAO inhibitors [6]. The pharmacological effect of tyramine arises from the selective inhibition of MAO-A towards MAO-B [7,8]. The selectivity of small molecule inhibitors of MAO-A and MAO-B was elucidated by resolving their three-dimensional structures co-crystallized with inhibitor [9]. Whereas a two-site cavity structure (entry and reactive site cavity) was identified for MAO-B, the active site of MAO-A is unique, shorter, and wider in comparison to the protracted and narrower substrate pocket in MAO-B [9]. The know-how generated from the elucidation of three-dimensional structures of the binding sites of MAOs has been applied for the design of new reversible and irreversible inhibitors, which have lately been employed for the treatment of affective and neurological disorders [10]. A number of reversible MAO-A inhibitors (RIMA) which lack the cheese effect were developed, among which only moclobemide was approved for the therapy of depression [10]. Clinically approved irreversible inhibitors of MAO-B for the treatment of Parkinson’s disease belong to several chemical classes, i.e., hydrazines, cyclopropylamines, and propargylamines. Three approved drugs, i.e., selegiline and rasagiline, enter in propargylamine class and are somewhat selective for MAO-B, while safinamide, a selective, reversible inhibitor, is used for the treatment of Parkinson’s disease [10]. However, most MAO inhibitors display low selectivity, which requires the steady development of novel selective inhibitors based on cumulative knowledge to generate rational design strategies. Since the lack of selectivity can cause adverse toxicities, the improvement of at-once detection of off-target interactions is extremely important to lower safety-related attrition drug rates before clinical testing [11,12]. The therapeutic capability of MAO’s inhibitors, in conjunction with an exhaustive knowledge of the 3D structure of the MAO-B active site, may contribute to significant improvement in the development of novel drugs for Parkinson’s disease. Recent updates on the design, synthesis, and clinical trials of new MAO candidates have been summarized in the paper of Baweja GS et al. [13]. Even though numerous compounds have been continuously synthesized and clinically tested as possible candidates for treating PD, the dietary restrictions and adverse effects developed urgently require new theoretical, experimental, and therapeutic strategies to overcome these shortcomings.

In this context, cheminformatics approaches are systematically used in the early stages of drug development projects [11,12,13,14,15,16,17,18,19,20,21,22] and environmental science to assist the experiment in order to expedite and save resources and costs [23,24,25,26,27,28]. In silico tools can contribute significantly to exploring side effects by handling off-target interactions, which necessitates a thorough knowledge of structural information about off-target proteins [29,30,31,32,33]. Theoretical and experimental studies included in the review of Baweja GS et al. [13] pointed out that minor structural changes could significantly or detrimentally influence the selectivity and inhibitory activity of various derivatives as potential MAO-B inhibitors. The surveyed SAR analyses [13] underlined the key role of various substituents on the basic skeleton of coumarin, chalcone, piperazine-linked pyridazinone, 1,3,4-oxadiazole, thiosemicarbazide, pyridoxine-resveratrol, methylthiosemicarbazone, benzylamine-sulphonamide, isoxazole carbohydrazide and safinamide derivatives as MAO-B inhibitors. For coumarin moiety-containing derivatives, the presence of hydroxy or halogen (Br, Cl) unit in position seven and halogen-substituted heterocyclics or substituted benzoxy unit increase the MAO-B activity [34,35,36]. The chalcone core-containing derivatives substituted on the A ring by the prenyl group of the methoxy group and the B ring by electron-withdrawing groups at the ortho position possess better MAO-B inhibitory activity [37,38]. Substitution at 4’position of pyridoxine-resveratrol derivatives improves the MAO-B activity as compared to 3′-substitution. The inclusion of sulfonamide and bezothiazole units at the benzylamine-sulphonamide derivatives skeleton improves the activity, while the NO2 electron withdrawing fragment decreases MAO-B activity. Likewise, the phenyl substituted at the piperazine core, the piperazine-linked pyridazine unit, and halogens contribute positively to MAO-B inhibitory activity.

Foray into molecular docking studies [35] revealed essential knowledge about the key interaction of potential MAO inhibitors and MAO enzyme active site residues, which are responsible for selectivity and inhibitory activity as follows: (i) coumarin-pyridazine derivatives [34] form hydrogen bonds with TYR:435, strong electrostatic interactions with GLN:206, TYR:326, and TYR:188, and hydrophobic contacts with PHE:168, LEU:171, ILE:199, ILE:316, and TYR:326 (ii) prenylated chalcones derivatives [37] realized hydrogen and hydrophobic interactions with TYR:398 and TYR:435, while dimethoxy-halogenated chalcone form hydrogen bond with TYR:326 and TYR:188, (iii) thiosemicarbazide derivatives [39] form van der Waals interactions with LEU:171, ILE:198, CYS:172, GLN:206, PHE:343, TYR:326, TYR:398, TYR:435, and FAD molecule as well as electrostatic interactions with GLN:65, TYR:188, CYS:172, ILE:198, TYR:326, GLN:206, and TYR:435, (iv) pyridoxine-resveratrol derivatives [40] establish hydrophobic interactions with ASN:181, CYS:323, ILE:180, ILE:207, ILE:325, ILE:335, LEU:97, PHE:108, PHE:208, PHE:352, TYR:69, TYR:407, TYR:444, VAL:210, and π-π stacking interactions with TYR:398. 

Picture drafted by the essential features of SARs and molecular docking approaches encouraged us to study a series of 126 MAO-A and B inhibitors in an effort to elucidate the structural elements and molecular interactions that control MAO-B potency and selectivity [5,41,42].

**Figure 1:**
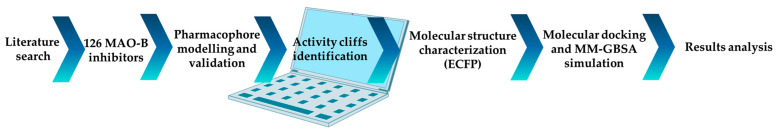
The workflow diagram used to assess the selectivity and potency of MAO-B inhibitors.

To reach these goals, we take advantage of a particular association of computational methods, including LBVS (ligand-based virtual screening) and SBVS (structure-based virtual screening), such as pharmacophore modeling, 3D atom-based QSAR, activity-cliff detection, extended-connectivity-fingerprints of diameter 4 (ECFP4) analysis, molecular docking, and MM-GBSA free-binding energies. The results obtained may contribute to the development of novel potent and selective MAO-B inhibitors with superior activity profiles aimed at fighting diseases caused by MAO-B. The workflow strategy is depicted in Figure 1.

## 2. Results and Discussion

### 2.1. Pharmacophore Modeling

A total of 45 pharmacophore hypotheses displaying 4, 5, 6, and 7 pharmacophoric points were generated, from which 27 hypotheses were externally validated by atom-based 3D QSAR adhering to conditions: Q^2^ > 0.6 and Pearson R > 0.8. Among all 45 pharmacophore models, particularly four-point pharmacophores registered the highest values of survival score, respectively, with better alignment of the active compounds (Table S1).

**Figure 2:**
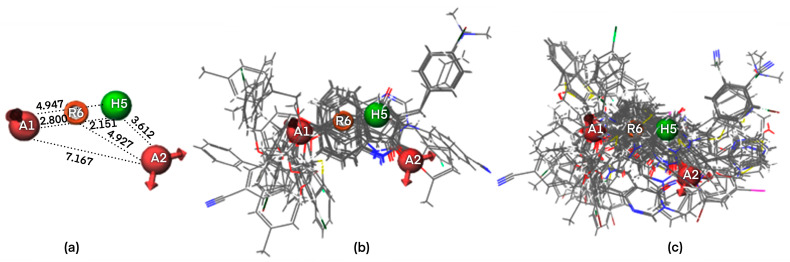
Pharm-1 inter-feature distances and putative interaction points: acceptor (A1, A2, pink), hydrophobic (H5, green), ring (R6 orange) superposed on the most selective compound **6**; the distances are reported in Angstroms (Å) (**a**). All selective MAO-B inhibitors (34 compounds) overlapped on Pharm-1 (**b**); All non-selective MAO-B inhibitors (92 compounds) overlapped on Pharm-1 (**c**).

**Figure 3:**
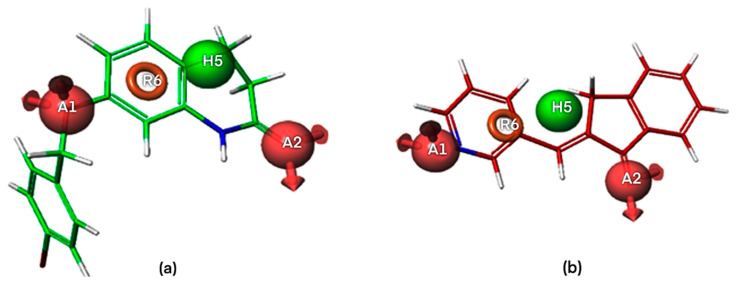
3D visualization of the most selective MAO-B inhibitor 6-[(4-bromophenyl)methoxy]-3,4-dihydro-1H-quinolin-2-one **6** (**a**) and the non-selective MAO-B inhibitor (2E)-2-(pyridin-3-ylmethylidene)-3H-inden-1-one **92** (**b**) aligned with the pharmacophoric sites of Pharm-1 hypothesis.

**Figure 4:**
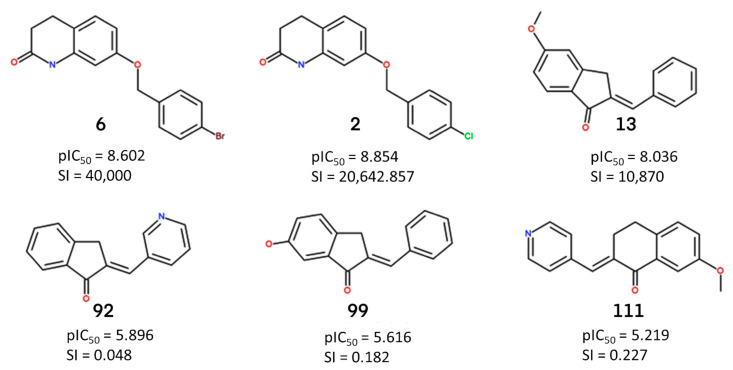
The structures, activities and selectivity index (SI) for the most active selective **6** (7-[(4-bromophenyl)methoxy]-3,4-dihydro-1H-quinolin-2-one), **2** (7-[(4-bromophenyl)methoxy]-3,4-dihydro-1H-quinolin-2-one), **13** ((2E)-2-benzylidene-5-methoxy-indan-1-one)) and non-selective **92** ((2E)-2-(3-pyridylmethylene)indan-1-one), **99** ((2E)-2-benzylidene-6-hydroxy-indan-1-one), **111** ((2E)-7-methoxy-2-(4-pyridylmethylene)tetralin-1-one)) MAO-B inhibitors.

The four-points pharmacophore (four-features) hypotheses were chosen for in-depth analysis in terms of atom-based 3D QSAR (Tables S1 and S2). One out of 27 four-points hypotheses, labeled AAHR.2 or Pharm-1, was selected as the best one, showing the largest adjusted survival score. AAHR.2 hypothesis consists of two hydrogen bond acceptors (A), one hydrophobic (H), and one aromatic ring (R). The inter-feature distances between pharmacophoric sites (A, A, H, R) of the Pharm-1 and the Pharm-1 hypothesis superimposed on the most selective compound **6** (pIC_50_ = 8.602, SI = 40,000) are drawn in Figure 2 and Figure 3a. For compound **6**, the two hydrogen bond acceptors correspond to the -O- atom linking the 3,4-dihydro-2(1H)-quinolinone ring system with substituent -CH_2_-C_6_H_4_-4I and (=O) group of the 3,4-dihydro-2(1H)-quinolinone ring, whereas the hydrophobe H5 correspond to -CH_2_- a unit of 3,4-dihydro-2(1H)-quinolinone ring. A distinct structural feature of several highly selective MAO-B inhibitors involved in pharmacophore modeling, i.e., compounds **6**, **2** (Figure 4), is the fused heteroaromatic ring which validates the presence of ring R6 in the Pharm-1 hypothesis.

#### 2.1.1. Pharmacophore Model Validation

The pharm-1 hypothesis was validated using the Phase methodology, including Fischer’s randomization test at a 95% significance level, scoring inactive, and generation of an atom-based 3D QSAR model. The most selective inhibitor, **6** (pIC_50_ = 8.602, SI = 40,000), fitted all four features, whereas the less potent and less selective compound **92** (pIC_50_ = 5.896, SI = 0.05) does not correctly fit the pharmacophore points R6 and H5 (Figure 3). Additionally, the training set of MAO-B inhibitors was mapped to the Pharm-1, revealing good alignments (Figure 2b,c). We can notice that selective MAO-B inhibitors show the appropriate alignment also between themselves, mainly in the central part of the molecule, which includes the fused ring core, displaying only low variations in the area occupied by the substituents. However, an irregular match with pharmacophore points and also among themselves was observed for non-selective MAO-B inhibitors in the central core and as well as for substituents (Figure 2c and Figure 3b). Therefore, a definite visual difference between selective and non-selective MAO-B inhibitors resulted from the alignment with the Pharm-1 hypothesis.

#### 2.1.2. Atom-Based 3D QSAR Approach

PHASE built 27 statistically valid 3D QSAR models (Q^2^ > 0.6) corresponding to the four features of pharmacophore models (Table S2). Of these, one atom-based 3D QSAR model associated with Pharm-1 was evaluated to determine its accuracy with regard to further virtual screening applications. The best atom-based 3D QSAR model, including 4PLS factors, is statistically robust, internally and externally (Table 1) as reflected by statistical parameters values: R^2^ = 0.900 (training set), Q^2^ = 0.774 (test set), Pearson’s R = 0.884 (test set). The correlation coefficient R^2^ of 0.900 suggested that the independent variable matrix (X) and dependent variable matrix (Y) are strongly correlated. The good predictive abilities of the model are supported by the Q^2^ > 0.6 and Pearson’s R > 0.8 values. The large value of the F test (167.200) and the very low value of the significance level of variance ratio *p* of 1.49 × 10^−36^ indicate a statistically significant QSAR model (Table 1). In summary, the developed atom-based 3D QSAR model is stable and predictive. The observed versus predicted biological activities for the training and test sets are plotted in Figure S1. For most of the MAO-B inhibitors, biological activities have been predicted with a good approximation. 

#### 2.1.3. Validation of Atom-Based 3D QSAR Model

The atom-based 3D QSAR model was validated using an array of techniques aiming at evaluating its robustness and predictive abilities. The values of the coefficients used for atom-based 3D QSAR model validation (Table 2) showed that statistical values fall within the recommended range: Squared Correlation Coefficient (R^2^) for fitting close to 1, Concordance Correlation Coefficient (CCC_test_) for training set > 0.85, Root-Mean-Square Errors (RMSE), and Mean Absolute Error (MAE) values close for both training and test sets, Squared Correlation Coefficient for prediction set > 0.6 and Q^2^_F1_, Q^2^_F2_, and Q^2^_F3_ values > 0.7. As derived from the residual, experimental, and predicted biological activity values, the atom-based 3D QSAR model is robust and predictive. Therefore, we can say that the atom-based 3D QSAR model is properly validated and can be used for further virtual screening experiments to extract promising lead molecules with inhibitory activity towards MAO-B.

**Figure 5:**
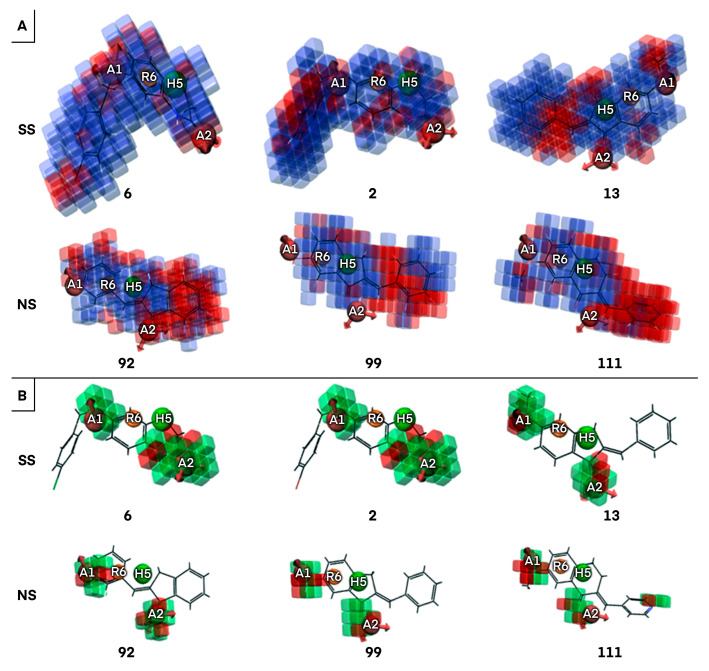
Representation of hydrophobic fields for most active and selective (SS) compounds (**6**, **2**, **13**) and less active and non-selective (NS) compounds (**92**, **99**, **111**) compounds (**A**); Electron withdrawing regions observed for most active and selective (SS) compounds (**6**, **2**, **13**) and less active and non-selective (NS) compounds (**92**, **99**, **111**) compounds (**B**). The most favorable features are depicted in blue and green (positive coefficients), whereas the detrimental features are shown in red (negative coefficients).

To prove the effective reliability of the atom-based 3D QSAR model, we aligned the ligand molecule on the Pharm-1 hypothesis and derived the atom-based field maps for QSAR visual analysis. The effects of electron-withdrawing and hydrophobicity characteristics of the three most active and selective (**6**, **2**, **13**) and the three less active and non-selective MAO-B inhibitors (**92**, **99**, **111**) on the biological activity are depicted in Figure 4 and Figure 5.

The analysis of the coefficients is helpful in labeling the properties of ligand structures that are likely to increase or decrease the activity. The most favorable pharmacophore fields are depicted in blue (hydrophobic field) and green (electron withdrawing) cubes and can be associated with increased activity, whereas the detrimental fields are depicted in red cubes displaying a negative influence on activity, according to the sign of their coefficient. This might give a clue regarding what functional groups are desirable or undesirable at certain positions in a molecule. Regarding hydrophobic fields, the maps are dominated by the positive coefficient in the case of most active and selective MAO-B inhibitors, with only several small red areas located mainly at the edges of compounds **6** and **2** and at the linker with p-hydroxy-phenyl substituent of **13** (Figure 5). The blue cubes located at the 3,4-dihydro-1H-quinolin-2-one core of compounds **6** and **2**, on the indanone ring of **13** (Figure 4), and also on several portions of the substituents attached to these rings illustrate that hydrophobic fields have a positive contribution on biological activity (Figure 5). Some positive hydrophobic areas are also located on substituents at positions 6 and 7 of 3,4-dihydro-1H-quinolin-2-one on compounds **6** and **2** and on phenyl substituent of **13,** which are confirmed by hydrophobic interactions observed for the co-crystallized ligand of MAO-B as well as by hydrophobic interactions resulted from docking (Docking section). Indeed, the (4-bromophenyl)methoxy substituent is present at position 6 or 7 on the 3,4-dihydro-1H-quinolin-2-one ring in the most active and selective MAO-B inhibitors **6** and **2** (Table S1). The extended red regions observed in the less active and non-selective MAO-B inhibitors, respectively, on the indanone ring (**92**), phenyl substituent (**99**), and pyridyl substituent (**111**) (Figure 5) indicate that the introduction of the nitrogen atom on these positions or the absence of the hydrophobic groups will negatively impact the compound potency. 

Visual analysis of Figure 5 reveals that the presence of larger areas defined by green cubes at the A1, and A2 sites, which corresponds to –OH group, -O-, =O, and N atoms in most active and selective MAO-B inhibitors (**6**, **2**, and **13**), pointing out the positive influence of electron-withdrawing properties for biological activity. Moreover, the attachment of suitable electron-withdrawing groups at these positions will potentiate MAO-B inhibition. On the contrary, the attachment of electron-withdrawing groups in the red areas will decrease inhibitory activity towards MAO-B. The positions of hydrogen bond acceptor sites on the main fused ring of selective MAO-B inhibitors (**6**, **2**), which are situated in the proximity of hydrogen bond acceptor sites of the Pharm-1, are different with reference to non-selective MAO-B inhibitors. In the case of less active and non-selective MAO-B inhibitors, the number of hydrogen bond acceptors is higher or lower (Figure 5), and they are placed further from pharmacophore sites (**92**, **99**, and **111**) compared to most selective MAO-B inhibitors (**6**, **2**, and **13**) (Figure 5). Most likely, in this area, the acceptor groups of the non-selective MAO-B inhibitors cannot interact with the binding site residues (Section 2.4). To further design MAO-B inhibitors, the green areas of the electron-withdrawing (aka H-bond acceptor) field and the blue areas of the hydrophobic field strongly advise that these features should be preserved and subsequent bioisosteric substitutions at these loci should be performed with substituents exhibiting equivalent properties (Figure 5). On the contrary, the substituents which are covered by red cubes on both fields have to be replaced or removed (Figure 5). This property field analysis provided deep insights into structural features crucial to the design of novel high-potency MAO inhibitors. In summary, we identified several common pharmacophore features of the most selective MAO-B inhibitors, which trigger maximal effects on biological activity and selectivity. Consequently, the Pharm-1 hypothesis validated by atom-based 3D QSAR can be confidently used to prioritize novel MAO-B candidates with increased susceptibility to selectively inhibit MAO-B targets.

### 2.2. Identification of Activity-Cliffs

**Figure 6:**
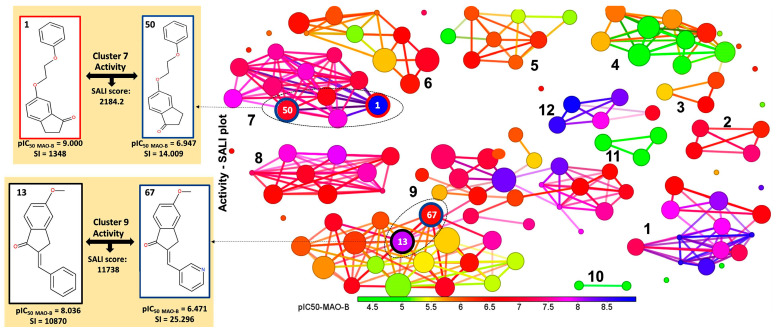
Activity cliffs network of 126 MAO-B inhibitors (the inhibitors shown as nodes whereas edges designate molecular similarity relationships; nodes are colored according to the compound’s affinity from green (lowest) to blue (highest). In addition, the size of the nodes is scaled according to their contribution to the local index.

The clustering based on the Flexophore descriptor identified 12 stable clusters, as shown in Figure 6, where the potency against MAO-B is depicted according to the color scale. The clustering of dataset compounds leads to the concentration of highly active MAO-B inhibitors inside clusters 1, 2, 7, 8, and 12, whereas clusters 8 and 12 contain exclusively selective compounds, and clusters 3, 4, and 6 include only non-selective inhibitors. The remaining compounds are dispersed among clusters 1, 2, 5, and 7 (Figure 6, Table S3). 

The activity and selectivity cliffs representing key disruptions in the structure-activity landscape of MAO-B inhibitors were identified on the basis of their SALI score. For example, compounds 1 and 50 were identified as an activity cliff with a SALI score of 2184.2, pIC_50_ ratios of 9/6.947, and SI ratios of 1.348/14.009, respectively. Inhibitors 1 and 50 belong to cluster 7, which are concentrated in several highly active compounds (11 structures). Regarding selectivity, cluster 7 consists mainly of moderately selective compounds, only compounds **1** and **54** display good selectivity (Table S3). Both MAO-B inhibitors possess a 2-methoxy-ethoxy benzene substituent, the structural difference being the position of the (=O) group of 2,3-didydroindene-one. This structural difference also leads to a significant decrease in SI, where compound **1** selectively inhibits MAO-B. Another selectivity cliff was observed for pairs **13** and **67** (pIC_50_ ratio of 8.036/6.471, SI ratio of 10870/25.296), compounds belonging to cluster 9, which is the largest, including **37** inhibitors. Cluster 9 contains highly and less potent MAO-B inhibitors with variable selectivity (Table S1). The explanation of divergent activity and selectivity of the compounds **13** and **67** arise from the structural difference consisting in the replacement of the phenyl ring of (2E)-5-methoxy-2-(phenylmethylidene)-3H-inden-1-one with pyridine. It seems that the introduction of a nitrogen atom into the aromatic six-membered ring, which increases the polarity and decreases the electron density in the ring, is not beneficial for potency and selectivity against MAO-B. A possible explanation could be the incapacity to form polar interactions with amino acid residues in close proximity.

### 2.3. ECFP (Extended-Connectivity Fingerprints) Analysis

**Figure 7:**
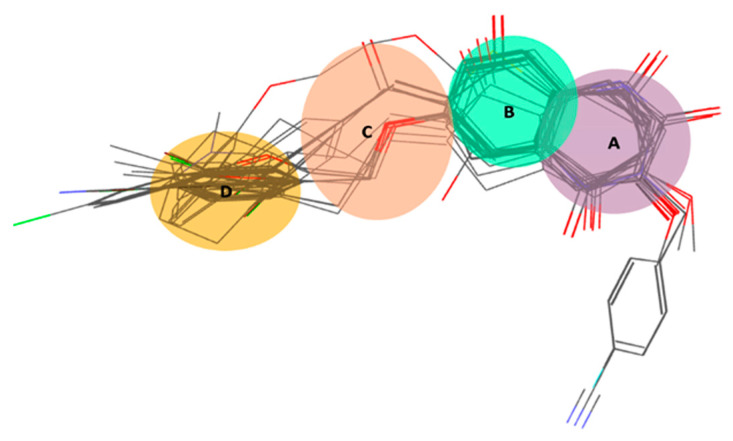
The 3D alignment of selective 126 MAO-B inhibitors resulted from ROCS overly [43,44] and the division of molecules in zones from A to D; the substructure areas are highlighted as follows: A—move, B—green, C—orange, and D—yellow.

The ECFP4 run on 126 MAO-B inhibitor structures generated 1024 bit-vectors that were further subjected to AUC analysis in conjunction with selectivity to MAO-B. As a result, a number of 268 significant AUC values were derived, of which 12 AUC values were greater than 0.9, 11 AUC values between 0.8 and 0.9, 28 AUC values between 0.7 and 0.8, and 47 AUC values between 0.6 and 0.7. Among these, we chose relevant substructures of radius 4 belonging to each molecular zone and shared by a minimum of 10% of MAO-B inhibitors, as shown in Figure 7. Their AUC values are listed in Table S5. The ring on the right side of the fused main core (Figure 7) was classified as category A. The structural difference between bitVector 623 (AUC = 0.962) and bitVectors 949 and 290 (AUC values of 0.444 and 0.351, respectively) (Table S5) consists in the absence of the NH group from the aromatic 6-membered ring and the replacement of the =O moiety attached to the neighboring carbon atom belonging to 3,4-dihydro-1H-pyridin-2-one with OH group. BitVector 623 is present in 9 out of 34 selective inhibitors, including the most selective **6**, **2**, and **13**, while in the non-selective ones, it is less frequently observed, only in 5 out of 92 compounds. The least active and non-selective inhibitors, **92**, **99**, and **111**, do not display this fingerprint, but all of them display bitVector 290. The presence of five ring substructures encoded by bitVector 3 (Table S5) is also favorable for selectivity, being present in the most selective MAO-B inhibitors **6**, **2**, and **13** having an AUC value of only 0.683, whereas bitVector 642 is present in both categories being not very discriminative (AUC = 0.675). The variance of substructures in zone B is reduced, and the phenyl ring linked to -O- is the most recurrent one. However, these substructures did not show any significant influence on the selectivity as the AUC values are close to random even though a relatively high number of compounds share these substructures 38, 33, and, respectively, 77. In zone C, the linker includes the substructure bitVector 478, which is also present in a large number of compounds (**52**), showing an AUC of 0.811 and implicitly favorable influence on selectivity. BitVector 478 is encountered in the most active and selective MAO-B inhibitors **6**, **2**, and **13** but absent in the less active and non-selective **92**, **99**, and **111**. A larger linker in zone C (bitVector 677) displays a moderate influence on selectivity with an AUC of only 0.603 being present in **3** selective MAO-B inhibitors, including compound **13**. The substructure encoded by the bitVectors 609 and 410, which describe a short linker between the fused core and substituent, shows very low AUC values of 0.425 and 0.383, respectively. BitVectors 609 is observed in non-selective compounds **99** and **111** negative influence on selectivity towards MAO-B. In zone D, four main substructures containing halogen substituents, such as Br and Cl bound to the phenyl ring (bitVector 910, bitVector 217), display AUCs of 0.664 and 0.645, showing little favorable influence on selectivity. BitVector 217 is found in the most selective MAO-B inhibitors **6**, **2**, and **13**, while bitVector 910 is only present in some compounds showing SI in the range 60–3000. The other substructures present in zone D are encoded by bitVectors 681 and 961, but none of them turned out to be relevant for selectivity since the AUC values are close to the random distribution. These results suggest that the substructures encoded by bitVector 623 in zone A and bitVector 478 in linker C contribute essentially to the MAO-B selectivity. Selective MAO-B inhibitors show an average of 2.47 bitVectors per molecule, whereas non-selective display an average of 1.12 bitVectors per molecule. We also identified the most frequent bitVectors in MAO-B inhibitors by adding the number of bits that were weighted by the number of instances in each selective and non-selective category, respectively. BitVectors 478, 623, and 642 were observed to be most frequent in selective MAO-B inhibitors with frequency values of 0.676, 0.264, 0.618 relative to non-selective MAO-B inhibitors, which display lower frequency values of 0.315, 0.054, and 0.293, respectively. 

### 2.4. Docking Analysis

**Figure 8:**
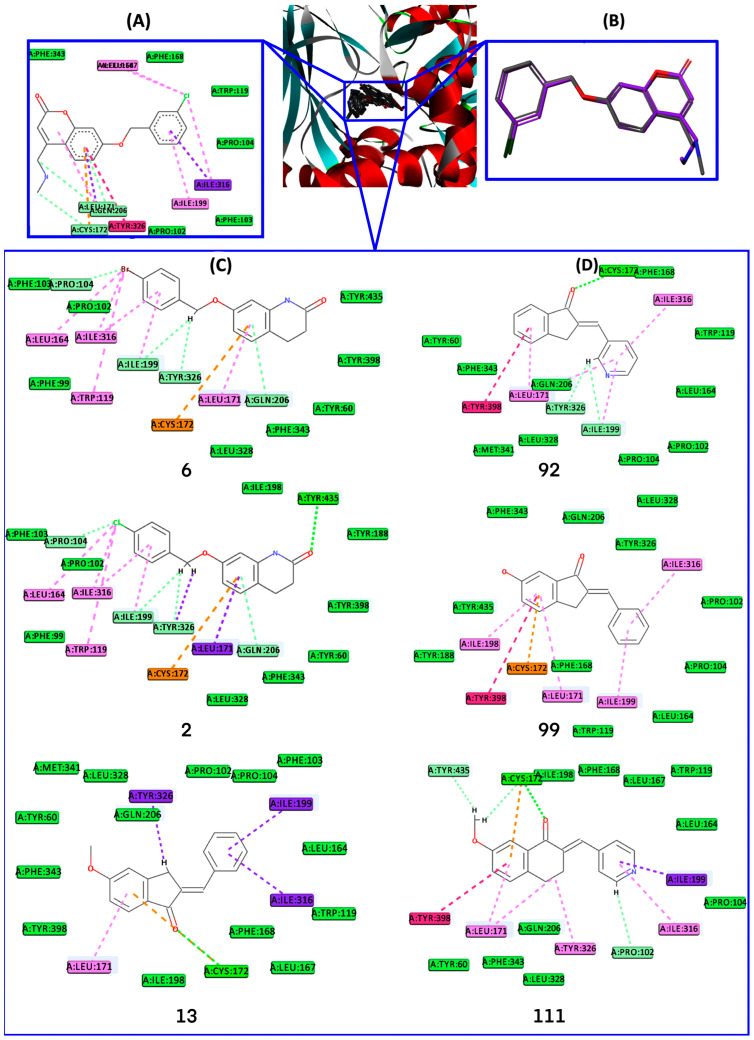
The docked pose of 7-(3-chlorobenzyloxy)-4-methylamino-methyl-coumarin (C18) (**A**) (grey), the overlay of RX structure (magenta), and best-docked pose of C18 (**B**), best-docked poses of most selective MAO-B inhibitors **6**, **2** and **13** (**C**), best-docked poses of non-selective MAO-B inhibitors **92**, **99** and **111** (**D**).

To investigate the interactions and conformational arrangement of MAO-B inhibitors within MAO-B active site, glide SP docking experiments were performed. The docking procedure was validated by portraying the interaction observed in 2V61 MAO-B X-ray crystal structure complexed with 7-(3-chlorobenzyloxy)-4-methylamino-methyl-coumarin (C18) and computing the RMSD between the coordinates of C18 co-crystalized and its best-docked poses. The best low energy docked pose of C18 in MAO-B (Figure 8) active site makes the following interactions: (i) 4-methylamino-methyl substituent: water hydrogen bond with HOH1388, and HOH1434, two carbon-hydrogen bond with CYS:172 and LEU:171; (ii) coumarin fused ring: π-sulfur and two carbon-hydrogen bonds with CYS:172, π-sigma, and π-alkyl with LEU:171, π-π T-shaped with TYR:326, π-donor hydrogen bond with GLN:206; (iii) phenyl ring of 3-chlorobenzyloxy substituent: two hydrophobe π-alkyl with ILE:199 and ILE:316; (iv) Cl atom: π-alkyl with LEU:164, LEU:167, and ILE:316. Since the RMSD between the coordinates of the RX co-crystalized ligand and the best-docked pose of C18 provided an RMSD value of 0.418 Å (Figure 8), we considered that the orientation of the RX ligand-receptor complex was reproduced with good accuracy. After demonstrating the validity of the docking protocol, the three most selective MAO-B inhibitors, **6**, **2,** and **13**, and three non-selective MAO-B inhibitors **92**, **99**, and **111** were docked into the MAO-B active site. For each inhibitor, binding capabilities inside the MAO-B binding site were assessed by: (i) selection of the best pose after performing detailed visual analysis of all five poses generated per ligand in comparison with the native ligand C18, and (ii) essential interactions with critical binding site residues PRO:102, LEU:171, CYS:172, ILE:199, GLN:206, ILE:316, TYR:326, TYR:398, TYR:435 [45,46,47].

The inhibitor MAO-B complexes, resulting from the docking procedure, were visually inspected by comparing the binding pattern and interactions of each inhibitor docked with that of the co-crystalized ligand C18 by employing Discovery Studio Visualizer facilities (Figure 8) [48]. Inspection of the binding position occupied by inhibitors inside of MAO-B active site indicated that the ligands are in the bipartite receptor binding bipartite cavity delineated by ILE:171, CYS:172, ILE:199, GLN:206, ILE:316, TYR:326, TYR:398, and TYR:435 residues, similarly with experimental observations of Binda et al. [45]. Besides contacts with these residues, additional interactions of MAO-B inhibitors with TRP:119, PRO:104, LEU:164, TYR:188, and PRO:104 were noticed. The best poses of selective inhibitors **6**, **2,** and **13** into MAO-B binding site registered the following interaction profile: compound 6: 3,4-dihydro-1H-quinolin-2-one makes H-bond with TYR:435, π-donor hydrogen bond with GLN:206, π-sulfur with CYS:172, π-alkyl with LEU:171, CH_2_ unit of (4-bromophenyl)methoxy- substituent makes a carbon-hydrogen bond with ILE:199 and TYR:326, π-alkyl with TRP:119 and ILE:316, alkyl with PRO:104, LEU:164, and ILE:316; compound **2** makes similar interactions with **6**, and two π–sigma interactions with LEU:171 and TYR:326, instead of π-alkyl and carbon-hydrogen bond with the same aminoacid residues; compound **13**: 5-methoxy-2,3-dihydroinden-1-one unit makes a conventional hydrogen bond with CYS:172, π-sulfur with CYS:172, π–sigma with TYR:326, and π-alkyl interaction with LEU:171, whereas phenyl ring substituent makes two π-sigma interactions with ILE:199 and another π-sigma interaction with ILE:316.

Non-selective MAO-B inhibitors interact with the MAO-B binding residues as follows: compound **92**: 7-methoxy-3,4-dihydro-2H-naphthalen-1-one unit makes a hydrogen bond with CYS:172, π-alkyl with LEU:171, π–π stacking with TYR:398, pyridine ring makes carbon-hydrogen bonds with LEU:199 and TYR:326, and three π-alkyl interactions with LEU:171, ILE:199, and ILE:316; compound **99**: 6-hydroxy-2,3-dihydroinden-1-one makes π–π stacking with TYR:398, π-sulfur with CYS:172, π-alkyl interactions with LEU:171, LEU:198, and phenyl substituent make π-alkyl interaction with ILE:199, and ILE:316; compound **111**: 7-methoxy-3,4-dihydro-2H-naphthalen-1-one core makes π–π stacking interaction with phenyl ring of TYR:398, hydrogen bond, carbon-hydrogen bond and π–sulfur interactions with CYS:172, alkyl interaction with LEU:171, π-alkyl interaction with LEU:171 and TYR:326, while pyridine substituent makes π–sigma interaction with ILE:199 and π-alkyl interaction with ILE:316, and carbon-hydrogen bond with PRO:102. The selectivity towards MAO-B arises not only from the position inside the binding pocket that all inhibitors occupy but also from the fact that selective inhibitors have more polar atoms that favor their orientation towards the hydrophilic area of the binding pocket and generate a large number of interactions with MAO-B residues, especially carbon-hydrogen bond with TYR:435 (compounds **6** and **2**) and π-donor with GLN:206 (compounds **6** and **2**) [45] (Figure 4 and Figure 8) which favors selectivity, while non-selective inhibitors make fewer interactions with the binding site residues and no hydrogen bonds with TYR:435 and TYR:326. Selective MAO-B inhibitors **6** and **2** interact with GLN:206 and TYR:326, relying on an unoccupied zone in the safinamide-MAO-B complex, which is similar to the experimental observations of Binda et al. [45]. The phenyl ring of 3,4-dihydro-1H-quinolin-2-one moiety is entangled by LEU:171 (sigma-π with MAO-B inhibitor **2**, π-alkyl with MAO-B inhibitor **6**), CYS:172 (π-sulfur) and GLN:206 (π-donor hydrogen bond). The presence of phenyl rings substituted with halogen (Cl, Br) as in MAO-B inhibitors **6** and **2** contributes to the stabilization of the ligand inside the active site of MAO-B by making multiple interactions as follows: phenyl ring sandwiches between ILE:199 and ILE:316 by π-alkyl interactions, whereas chlorine and bromine make halogen alkyl interactions with PRO:104, LEU:164 and ILE:316, carbon-hydrogen bond with PRO:104 and π-alkyl interaction with TRP:119. It is well acknowledged that experimental data demonstrated the powerful influence of halogen bonds on binding affinity, i.e., Rodríguez-Enríquez et al. [34] observed that the selectivity of coumarin derivatives is increased when bromine atom was present at the C7 position of coumarin fragment. The H-bonds and hydrophobic interactions observed hold ligands inside the binding site and inherently stabilize their orientation. Moreover, the presence of π–π stacking, H-bonds, and hydrophobic interactions release the possibility of not developing resistance to mutations. It is recognized that the deficit of π–π stacked interactions can trigger negative consequences on inhibitory activity. Hence, a strong hydrogen bond of 2.435 Å (MAO-B inhibitor **6**) and 2.362 Å (MAO-B inhibitor **2**) with selective residue TYR:435 can contribute substantially to selectivity than a weaker one, which can be rearranged energetically in concurrent MAO-A isoform or closely similar enzymes [49]. The results obtained in the current paper are consistent with previous experimental investigations [45]. More detailed information on the established interactions of the ligands with the active site residues and their lengths is provided in the Supplementary Information (Table S6). Moreover, ECFP4 bitVector 623 encodes the substructure present in 3,4-dihydro-1H-quinolin-2-one, which makes hydrogen bond interaction by means of =O with -OH group of TYR:435, which correlates the insight resulting from two different computational methods (Table S3). This docking study complements the results obtained from pharmacophoric 3D QSAR and ECFP4 analyses. 

### 2.5. MM-GBSA Binding Free Energy Analysis

**Figure 9:**
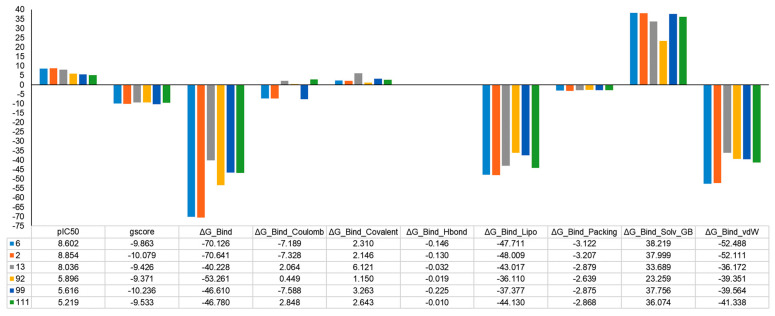
The pIC_50_, the docking scores, the total free binding energies, and the energy components of the six ligand-receptor complexes (kcal/mol).

Often, the employment of molecular docking and MM-GBSA has turned out to be a promising strategy to correctly identify binding poses and reliably rank the ligands [50]. In this context, the protein–ligand affinities were evaluated by calculating the MM-GBSA binding free energy (Figure 9). Among the six studied protein–ligand complexes, the **2**-MAO-B and **6**-MAO-B complexes exhibited the most negative values, which indicates a stronger binding affinity (−70.64 kcal/mol and −70.12 kcal/mol) comparable with the other complexes, while for the **13**-MAO-B complex was much lower (−40.22 kcal/mol). Furthermore, the assessment of the dissociation energy components emphasized the major contribution of non-polar solvation (DGbind Lipo) and the van der Waals interactions (DGbind vdW) in the stability of docked complexes and ligands binding affinities, especially for **2-** and **6**-MAO-B complexes (Figure 9). It was also observed that for all complexes, both DGbind covalent and DGbind solv GB components delivered unfavorable energies for ligand binding. The unexpected DGbind value of the **13**-MAO-B complex is influenced by the detrimental contribution of the DGbind covalent, DGbind Coulomb, and DGbind Solv GB components. These may suggest that some residues may undergo important rearrangements in the active site, and the corresponding conformational penalty is not accounted for. The MM-GBSA binding energies are in consensus with and complementary to docking scores and experimental findings.

The present study provides a reliable theoretical basis for future structural optimization, design, and synthesis of MAO-B inhibitors with improved properties and activity profiles.

## 3. Materials and Methods

### 3.1. Dataset

**Figure 10:**
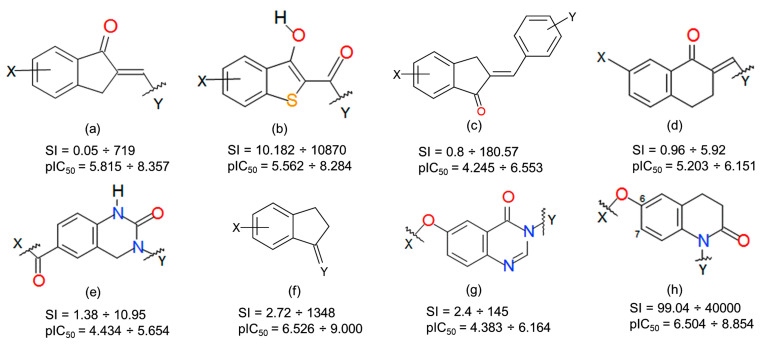
The templates of data set MAO-A and B inhibitors, their potency (pIC_50_)_,_ and selectivity index (SI) are calculated as the ratio between IC_50_ for MAO-A and IC_50_ against MAO-B; heteroarylidene-1-indanone (**a**), 2-benzylidene-1-indanone (**b**), benzo[b]tiophen-3-ol (**c**), 2-heteroarylidene-1-tetralone derivatives (**d**), C6-and N1-substituted 3-methyl-3,4-dihydroquinazolin-2(1H)-one (**e**), indanone (**f**), N3/C6-disubstituted 4(3H)-quinazolinone (**g**), and C6- and C7-substituted 3,4-dihydro-2(1H)-quinolinones (**h**).

The pharmacophore hypothesis and atom-based 3D-QSAR model were generated using a series of 126 derivatives comprising heteroarylidene-1-indanone [51] (Figure 10a), 2-benzylidene-1-indanone [52] (Figure 10b), benzo[b]tiophen-3-ol [53] (Figure 10c), 2-heteroarylidene-1-tetralone derivatives [54] (Figure 10d), C6-and N1-substituted 3-methyl-3,4-dihydroquinazolin-2(1H)-one [55] (Figure 10e), indanone [56] (Figure 10f), N3/C6-disubstituted 4(3H)-quinazolinone [57] (Figure 10g), and C6- and C7-substituted 3,4-dihydro-2(1H)-quinolinones [58] (Figure 10h) displaying a wide range of biological activities and selectivities, assessed by biological methods (Table S3).

The biological activities were determined against “single protein”, whereas standard relations “=“ and “<” for MAO-A, and “=” for MAO-B were retained. The molecular structures of 126 MAO-A/MAO-B inhibitors were sketched, saved as isomeric smiles strings using MarvinSketch of ChemAxon’s suite, and checked for duplicates prior to processing. The canonical smiles of the 126 MAO inhibitors are shown in Table S3. The experimental IC_50_ values were converted to a logarithmic scale (pIC_50_ = −logIC_50_) prior to pharmacophore modeling and atom-based 3D QSAR analyses. The distribution of biological activities is normal, spanning over 4.76 logarithmic units (Figure S2a,b). The selectivity index (SI) of 126 compounds was calculated employing potency ratios, respectively half inhibitory concentrations (IC_50_), against MAO-B and MAO-A isoforms: SI = IC_50_MAO-A/IC_50_MAO-B. Thus, the pIC_50_ and SI range for selective ones is between 6.072 ÷ 9.000 and 57 ÷ 40,000, respectively, while non-selective ones are between 4.245 ÷ 8.699 and 0.048÷49.032, respectively. Overall, the dataset of MAO-A and B inhibitors includes 34 selective MAO-B inhibitors showing 11 distinct Bemis–Murko frameworks (BMF) [59], whereas 92 non-selective display 13 unique BMF. The charges and stereochemistry were systematized with the help of Instant JChem (Instant JChem, v. 5.12.4, 2013) of the Chemaxon suite [60]. The values of drug-like descriptors were calculated with the help of FILTER software (FILTER, OpenEye Scientific Software, Santa Fe, NM, USA. http://www.eyesopen.com) (Figure S3). The MAO-A and B inhibitors fulfill drug-like conditions by compliance with Lipinski’s criteria [61] as follows: HBA = 0 ÷ 4, HBD = 0 ÷ 2, MW= 209.24 ÷ 594.18, RBN = 1 ÷ 5, XLogP = 0.52 ÷ 6.21, Polar Surface Area 2dPSA = 9.23 ÷ 91.7 (Figure S3).

### 3.2. Pharmacophore Modelling

The default setting of PHASE having hydrogen bond donor (D), hydrogen bond acceptor (A), hydrophobic (H), negative ionic (N), positive ionic (P), and aromatic ring (R) [62,63] features was used to derive pharmacophore hypotheses through “Develop Pharmacophore Model”. The phase includes the structure preparation step (LigPrep, https://www.schrodinger.com/), which adds hydrogens, converts 2D to 3D structures, yields stereoisomers, neutralizes charged structures, enumerates tautomers and ionization states in the pH range of 7.2 ± 0.2. The prepared ligands undergo conformational search using ConfGen (ConfGen, Schrödinger, LLC, New York, NY, USA, 2018) [64] to generate a maximum of 100 conformers per rotatable bond and 1000 conformers per compound, respectively. All generated conformers were subjected to energy minimization using the optimized potentials for liquid simulations (OPLS)-2005 semi-empirical force field [65]. The conformers were confined within an energy window of 50 kJ/mol [45], while irrelevant conformations were discarded, setting the heavy atom’s root mean squared deviation (RMSD) at 2 Å. All 126 MAO-A and MAO-B inhibitors were aligned over 3D conformation of the most selective MAO-B inhibitor 6 (Table S3), showing pIC_50_ = 8.602 and SI = 40,000. Thus, 11 most active (pIC_50_ = 7.000 ÷ 9.000) and eight less active (pIC_50_ = 4.245 ÷ 4.690) inhibitors of MAO-B (Table S3) were used to develop the pharmacophore model. The survival score (S) was used to detect the best hypothesis from the engendered models and allocated an overall ranking of all hypotheses [62]. The atom-based 3D QSAR models were constructed for all valid pharmacophore models involving randomly built binary training/test sets, where the grid spacing was set to 1.0 Å. Subsequently, a rigorous analysis of the scores and alignment of the selective ligands to the resulting pharmacophore hypotheses was carried out, allowing the selection of the best hypothesis. The assessment and validation of a pharmacophore hypothesis are required; hence, the best pharmacophore model Phar-1 was validated by Fischer’s randomization test at a 95% significance level, scoring inactive, and atom-based 3D QSAR model.

### 3.3. Atom-Based 3D QSAR

PHASE module from Schrödinger enables the construction of atom-based 3D QSAR models using ligands activities that match the acquired pharmacophore hypothesis. Valid pharmacophore hypotheses necessitate compliance with statistical criteria and must be rigorous to recognize the active compounds. Hence, the Pharm-1 hypothesis was engaged to overly MAO-B inhibitors aiming at developing an atom-based 3D-QSAR. Atom-based 3D-QSAR procedure involves the description of the molecules as overlapping van der Waals spheres that are allocated to one of the pharmacophore hypotheses [62]. The absence of any additional data is required to perform statistical validation by splitting the data set into training and prediction sets (for model predictive assessment) using random division [66,67]. The training set includes 70% of randomly selected MAO-B inhibitors representing 89 compounds, and the test set includes 30% of MAO-B inhibitors representing 37 compounds that preserve a similar distribution of biological activity. The distribution diagrams of the biological activities against MAO-A and MAO-B were plotted (Figure S2a,b). While developing an atom-based 3D-QSAR model, the van der Waals spheres of the aligned training set of MAO-B inhibitors were placed into a standard grid of cubes. Each cube is ascribed zero or as many as 6 “bits” in order to record the different types of atoms included in the training set which occupy the same region in space. This mapping generates binary-valued models that are considered independent variables to build partial least squares (PLS) atom-based 3D QSAR models. The atom-based 3D-QSAR model was built by applying a maximum of four PLS factors because further increment of the PLS factors was not accompanied by an enhancement of statistical parameters or predictive abilities. Internal and external validation parameters for atom-based 3D QSAR models provided by PHASE include the squared correlation coefficient for fitting (Equation (S1) in Table S4), the squared correlation coefficient for the test set (q^2^), standard deviation (SD), Pearson’s correlation coefficient (Pearson’s R for the test set), statistical significance (P), and Fisher’s test (F). In the context of predictive QSAR modeling, besides validation carried out by PHASE, we also tested the atom-based 3D QSAR model for predictivity, aiming to demonstrate the statistical quality, including internal and external predictive power. Hence, the most relevant statistical parameters [67] used for internal (Equations (S1)–(S5)) and external (Equations (S6)–(S10)) validation of atom-based 3D QSAR model (Table S4) were: $r^{2}$- Squared Correlation Coefficient for fitting; values close to 1 indicate a strong relationship between the dependent and the independent variables; $r_{pred}^{2}$—Squared Correlation Coefficient for prediction set [68], values greater than 0.6 are considered acceptable; q—Leave-n-Out Correlation Coefficient [69], values greater than 0.7 are adequate; $Q_{F1}^{2}$ [70], $Q_{F2}^{2}$ [71], $Q_{F3}^{2}\;$ [72] are similar to q, where values greater than 0.7 are considered sufficient; $RMSE_{tr}\;$ and $\;RMSE_{pred}$—root-mean-square errors for the training and prediction set, which have to display comparable values [73]; $MAE_{tr}\;$ and $MAE_{pred}$—mean absolute error for the training and prediction set, whose values have to be similar [74]; $CCC_{tr}\;$ and $CCC_{pred}$—Concordance Correlation Coefficient for the training and prediction set [75], which have to show values greater than 0.85.

### 3.4. Activity Cliffs

One of the main challenges in drug design is understanding the relationship between molecular descriptions and the appropriate biological activity. This relationship is influenced by small structural changes which alter the biological activity. Activity cliff (AC) captures these chemical modifications playing a key role in biological activity establishment. AC is generally defined by a pair of structurally similar compounds showing large differences in their potency towards the same protein target [76]. The AC discontinuities [77] provide essential SAR information by detection of slight structural alterations crucial for the biological activity of compounds, such as “magic methyl” effects [78] which are of great relevance during the early stages of SAR optimization. Small structural alterations may arise in considerable differences in potency originating from various causes: steric clashes with the biological receptor, reduced/increased number of H bond donors/acceptors, changes in ligand conformation, etc. Therefore, ACs should be systematically identified by a common computational and medicinal chemistry effort to support compound optimization [79]. To accomplish this complex task, molecular similarity, potency anomalies, and experimental accuracy have to be carefully assessed since, in some cases, medicinal and computational chemistry provide different views [77]. In this work, we aim to identify activity cliffs in the landscape of the current dataset of MAO-B inhibitors for a detailed understanding of the SAR landscape. In this analysis, we present the sensitive SAR features which are responsible for the strong inhibition of the MAO-B. The relevance of the current study consists in the detection of structural features which may infer particular pharmacophoric characteristics to assist the further design of novel potent inhibitors against MAO-B. Hence, we identified and characterized activity cliffs by means of the structure-activity landscape index (SALI) [80] calculated with OSIRIS DataWarrior 2.5.2 [81] software based on the Flexophore descriptor [82].

$$SALIi,j=\frac{\left|Ai-Aj\right|}{1-sim\left(i,j\right)}$$

where *Ai* and *Aj* represent the activity of compounds *i* and *j* and *sim* designates the similarity between compounds *i* and *j*.

### 3.5. Extended-Connectivity Fingerprints (ECFPs)

ECFPs are circular fingerprints showing multiple useful qualities: fast calculation, are not predefined, and can depict an infinite number of various molecular features (e.g., stereochemical information). Moreover, they are less computationally demanding and allow easy interpretation of the results [83]. ECFPs are widely used in computational chemistry, especially for molecular structure characterization, chemical space analysis, clustering, structure-activity modeling, etc. The ECFPs are very suitable in similarity search for the recognition of the presence or absence of specific substructures; therefore, ECFPs are frequently involved in drug discovery to discriminate between active and inactive compounds [84]. ECFPs are derived using a variant of the Morgan algorithm [83], which was initially suggested as a method for resolving the molecular isomorphism problem [85].

Pursuant to the molecular group localization, these pivotal substructures were divided into five zones, A to E, and mapped on the overlaid molecular scaffold of MAO-A and MAO-B inhibitors (Figure 7) after performing 3D superposition with ROCS v.3.2.1.4 software [35,36]. In each area from A to E, we identified the fingerprints which correspond to molecular substructures positioned in the respective area and listed the most relevant ones in Table S5. The dataset of MAO-B inhibitors was split into 34 selective and 92 non-selective, which were explored by means of ECFP4 to identify key substructures which are prevalent in the selective with respect to non-selective. The evaluation of the results was carried out by means of the area under the receiver operating characteristic curve (AUC), which is a well-known parameter related to the discriminative ability toward the whole ranking list [86].

### 3.6. Docking

Molecular docking is relevant for drug design because of the accurate prediction of the experimental interaction mode and estimation of the binding affinity of the ligand in the corresponding protein target binding site. Molecular docking using the Glide module from the Schrödinger suite [87,88,89] was applied to predict binding modes, including conformational orientation and positions of the dataset ligands with MAO-B. The docking procedure was carried out through standard prediction (SP) docking mode involving default settings since this procedure is appropriate to dock a large number of ligands of unknown quality. The docking methodology engaged the rigid treatment of the receptor, whereas the ligands were considered flexible in order to attain the most favorable interaction pattern with binding site residues.

The X-Ray structures of human MAO-B were fetched from the RCSB Protein Data Bank (http://www.rcsb.org, accessed on 27 April 2022) and the structure with the highest resolution (PDB ID: 2V61, resolution: 1.70 Å, R-Value Free: 0.201) bound to 7-[(3-chlorobenzyl)oxy]-4-[(methylamino)methyl]-2h-chromen-2-one (C18) inhibitor, which display significant 3D similarity, in terms of Tanimoto Combo = 1.33, Combo Score = 1.5, Shape Tanimoto = 0.81, with the most selective MAO-B inhibitor, 6, was selected.

Protein Preparation Wizard [90] in Maestro [91] ensures preparation and refinement of the receptor using tools that allow (i) the rectification of multiple bonds, hydrogen atoms addition, merging non-polar H, and clearance of water molecules situated beyond 5 Å from the ligand, metal ions and cofactor; (ii) the assignment of protonation states for ASP, GLU, and/or tautomers for HIS, as well as the optimization of hydroxyl groups positions to maximize the hydrogen-bonding network; (iii) processing of protein structure to identify and adjust the current errors (e.g., incomplete residues, lacking side loops/chains, errors regarding protonation states of ASP, and GLU and also the tautomers of HIS, and/or misoriented amide units of ASN and GLN residues); (iv) the configuration of salt bridges; and (v) the refinement of protein–ligand complex by means of a successive restrained minimization involving the OPLS_2005 force field with an RMSD threshold of 0.3 Å [87,88,89]. Neutral and protonated states of HIS, ASP, and GLU were also sampled along with the two HIS tautomers (proton on either the Nd or Ne nitrogen). To illustrate the shape and properties of the receptor, the grid box of the 2V61 X-ray complex was created via Maestro’s receptor grid generation option using the co-crystallized ligand as the central box point to define the active site. The ligands as prepared for the pharmacophore step were employed in docking simulations. The best docking pose among the five generated poses for each ligand was chosen by analyzing its interactions with protein target MAO-B. The docking procedure was validated by (i) redocking the crystallized ligands (C18) of MAO-B and (ii) computing RMSD between the crystal ligand (designated as reference) and the predicted structures using the Superposition option in the Schrödinger Maestro module [91].

### 3.7. MM-GBSA Binding Free Energy

Molecular Mechanics—Generalized Born Surface Area (MM-GBSA) included in the Schrödinger Prime module [92,93] is often employed to evaluate docking poses, determine structural stability, and assess the free binding energy of the ligand–protein complexes [50]. The complexes were minimized using the OPLS 2005 force field and the following thermodynamic and desolvation parameters were explored: binding energy (DGbind), solvation model (DGbind Coulomb), non-polar solvation term (DGbind Lipo), hydrogen-bonding correction (DGbind Hbond), covalent binding (DGbind Covalent), π–π packing correction (DGbind Packing), generalized Born electrostatic solvation energy (DGbind Solv GB), and van der Waals interaction (DGbind vdW).

## 4. Conclusions

In the current study, we developed a reliable computational workflow involving pharmacophoric atom-based 3D QSAR, ECFP analysis, clustering, detection of activity cliffs, docking, and MM-GBSA free binding energies to identify the key structural features responsible for potency and selectivity of a dataset of 126 MAO-B inhibitors. The four-point pharmacophore hypothesis, Pharm-1, including two hydrogen bond acceptors (A), one hydrophobic group (H), and one aromatic ring (R), was the best among all 27 hypotheses validated by atom-based 3D QSAR and had the best survival score. The atom-based 3D QSAR developed on the basis of the Pharm-1 hypothesis was manifold validated, showing good fitness with the experimental data. In addition, hydrophobic and electron-withdrawing field maps of the atom-based 3D-QSAR revealed the relationships between structural features and inhibitory activity. To further design superior MAO-B inhibitors, the green areas at the A1 and A2 sites associated with the electron-withdrawing field (e.g., the –OH unit, -O-, =O, and N atoms on the most three active /selective (**6**, **2**, and **13** compounds) together with the positive sign of the coefficients and the blue areas of the hydrophobic field (e.g., located on the 3,4-dihydro-1H-quinolin-2-one (**6**, and **2**) and indanone (**13**) cores of the most selective compounds) strongly recommend that these features should be preserved and further bioisosteric substitutions are mandatory to positively impact the biological activity and selectivity over the MAO-B. On the contrary, the attachment of electron-withdrawing units in the red areas will negatively impact the inhibitory activity towards MAO-B. The positive influence of these fields on the MAO-Bs selectivity and activity is consistent with the hydrophobic interactions made by co-crystallized ligand C18 as well as the hydrophobic interactions resulting from the docking approach.

The ECFP4 analysis highlighted the substructure encoded by bitVector 623 (e.g., 3,4-dihydro-1H-quinolin-2-one core) in zone A and bitVector 478 in the linker C as essential for MAO-B selectivity, being present in the most selective MAO-B inhibitors (**6**, **2**, and **13**) and absent in non-selective ones (**92**, **99**, and **111**). The BitVectors 478, 623, and 642 were also observed to be the most common in selective MAO-B inhibitors. Clustering analysis provided a good separation of active/potent with respect to less active MAO-B inhibitors. Two activity cliffs consisting of exclusively selective ligands were detected across the chemical space of 126 MAO-B inhibitors. The docking outcomes showed that TYR:435, TYR:326, CYS:172, and GLN:206 residues involved in essential hydrogen bonding and π-donor interactions stabilize the selective ligands (e.g., **6**, **2**, and **13**) in the MAO-B active site and favor selectivity over MAO-B. In contrast, non-selective ligands make only fewer interactions with the active site residues and no hydrogen bonds with TYR:435 and TYR:326, which are acknowledged to be vital for selectivity and catalytic activity toward MAO-B [94]. Furthermore, the presence of phenyl rings substituted with halogen (Cl, Br) significantly strengthens the ligand inside MAO-B, the active site, by forming multiple interactions with ILE:199 and ILE:316, PRO:104, LEU:164, and TRP:119. Atom-based 3D QSAR, ECFP4, docking, and MM-GBSA analysis provided consistent outcomes, underlining the essential contribution of 3,4-dihydro-1H-quinolin-2-one scaffold to potency and selectivity, as well as the relevance of the halo aryl substituent in stabilizing the interaction with MAO-B. In our future endeavors, experimental studies will be considered to validate these findings. Importantly, readers will gain knowledge of key structural features essential for MAO-B activity. This computational scenario may draw more attention to the design of novel, potent, and selective MAO-B inhibitors using other chemical databases or be used to search hit molecules for other targets involved in appropriate diseases.

## Figures and Tables

**Table 1:** The statistical parameters of the atom-based 3D QSAR model.

PLS Model for Pharm-1				
Statistical parameters	#1	#2	#3	#4
% of molecules in the training set	70	70	70	70
% of molecules in the test set	30	30	30	30
Training set, r^2^	0.592	0.794	0.851	0.900
Test set, q^2^	0.624	0.665	0.777	0.774
Pearson correlation coefficient (Pearson-R)	0.807	0.835	0.902	0.884
Stability	0.898	0.811	0.772	0.736
Standard deviation (SD)	0.752	0.537	0.461	0.381
Variance ratio (F-value)	113.200	148.700	144.500	167.200
Significance level of variance ratio (*p*-value)	7.57 × 10^−17^	3.61 × 10^−27^	2.55 × 10^−31^	1.49 × 10^−36^

**Table 2:** Validation of atom-based 3D QSAR model.

Pharm-1	CCC_tr_	CCC_test_	Q^2^_F1_	Q^2^_F2_	Q^2^_F3_	R^2^_pred_	RMSE_tr_	MAE_tr_	RMSE_test_	MAE_test_
M1	0.947	0.869	0.774	0.774	0.794	0.861	0.369	0.275	0.527	0.456

## Data Availability

Data are contained within the article and Supplementary Materials.

## Supplementary Materials

The following supporting information can be downloaded at: https://www.mdpi.com/article/10.3390/ijms24119583/s1.

## Glossary

A: acceptors; AC, Activity cliff; AUC, Area Under the Curve; BMF, Bemis Murcko Framework; ECFPs, Extended-Connectivity Fingerprints; H, hydrophobic; HBA, Hydrogen Bond Acceptor; HBD, Hydrogen Bond Donor; LBVS, Ligand Based Virtual Screening; MAO-A, Monoamine Oxidase A; MAO-B, Monoamine Oxidase B; PDB, Protein Data Bank; QSAR, Quantitative Structure–Activity Relationship; R, aromatic rings; RBN, rotatable bond; RMSD, Root Mean Squared Deviation; ROCS, Rapid Overlay of Chemical Structures; SBVS, Structure-Based Virtual Screening; VS, Virtual Screening.
