# Supplementary material for: New Insights on the Activity and Selectivity of MAO-B Inhibitors through In Silico Methods
Source: Int J Mol Sci. 2023 May 31;24(11):9583. doi: 10.3390/ijms24119583 (PMC10253494; doi:10.3390/ijms24119583)
Supplement: Supplementary file 1 [file ijms-24-09583-s001.zip › ijms-2402606-supplementary.pdf]

International Journal of
Molecular Sciences

MDPI

Supplementary Material

New Insights on the Activity and Selectivity of MAO-B Inhibitors through In Silico Methods

Liliana Pacureanu †, Alina Bora † and Luminita Crisan *

"Coriolan Dragulescu" Institute of Chemistry, 24 Mihai Viteazu Ave., 300223 Timisoara, Romania; pacureanu@acad-icht.tm.edu.ro (L.P.); alina_bora@acad-icht.tm.edu.ro (A.B.)
* Correspondence: lumi_crisan@acad-icht.tm.edu.ro
† These authors contributed equally to this work.

Table S1. Statistical details of the best pharmacophore hypotheses with four features

ID	Survival	Post-hoc	Site	Vector	Volume	Selectivity	# Matches	Activity	Inactive
AARR.17	3.143	3.143	0.680	0.747	0.714	1.196	9	8.854	1.315
ADHR.9	3.349	3.349	0.700	0.943	0.707	1.335	8	8.854	1.743
**AAHR.2**	**3.172**	**3.172**	**0.630**	**0.949**	**0.593**	**1.269**	**8**	**8.602**	**1.546**
ADHR.2	3.177	3.177	0.640	0.953	0.589	1.313	8	8.602	1.305
AADH.3	3.195	3.195	0.700	0.939	0.559	1.053	8	8.602	1.398
AADR.3	3.312	3.312	0.660	0.976	0.676	0.992	9	8.602	1.456
ADHR.1	3.497	3.497	0.940	0.992	0.569	1.257	8	8.602	1.611
ADHR.3	3.100	3.100	0.620	0.925	0.553	1.328	8	8.268	1.509
AADR.4	3.173	3.173	0.580	0.970	0.625	1.018	9	8.268	1.748
AAHR.3	2.489	2.489	0.220	0.734	0.531	1.327	8	7.959	1.482
AHRR.24	3.089	3.089	0.540	0.823	0.721	1.468	8	7.770	1.368
AAHR.5	3.258	3.258	0.590	0.912	0.755	1.352	8	7.745	1.648
ADHR.7	3.412	3.412	0.730	0.960	0.722	1.333	8	7.745	1.699
ADRR.13	3.170	3.170	0.610	0.827	0.731	1.207	9	7.745	1.709
AARR.15	3.240	3.239	0.620	0.872	0.748	1.180	10	7.745	1.837

AHRR.19	2.802	2.802	0.420	0.808	0.572	1.395	8	7.602	1.362
ADRR.15	2.620	2.620	0.380	0.695	0.544	1.214	9	7.602	1.650
ADRR.3	2.756	2.756	0.380	0.818	0.559	1.215	9	7.328	0.880
AADR.7	2.659	2.659	0.420	0.722	0.515	1.049	9	7.328	1.121
AARR.41	2.705	2.705	0.320	0.746	0.642	1.204	10	7.328	1.505
AADR.12	2.690	2.690	0.400	0.869	0.424	1.041	9	7.328	1.691
ADRR.17	2.580	2.580	0.380	0.689	0.508	1.221	9	7.328	1.716
ADRR.12	3.026	3.026	0.550	0.858	0.621	1.201	9	7.328	1.722
ADHR.17	3.146	3.146	0.570	0.857	0.724	1.393	8	7.009	1.122
AADH.4	3.129	3.129	0.660	0.920	0.553	1.072	8	7.009	1.632
ADRR.6	3.211	3.211	0.680	0.815	0.717	1.186	9	7.009	1.836
AARR.14	3.139	3.139	0.560	0.871	0.707	1.182	10	7.009	1.963

Table S2. Statistical parameters of the best atom-based 3D QSAR corresponding to the pharmacophore hypotheses with four features

ID	# Factors	P	SD	R-squared	F	Stability	RMSE	Q-squared	Pearson-R	Activity	SI
AADH.3	4	3.42E-30	0.351	0.932	180.500	0.741	0.697	0.681	0.856	8.602	40000.000
AADR.3	4	2.77E-32	0.433	0.872	126.400	0.807	0.644	0.712	0.860	8.602	40000.000
**AAHR.2**	**4**	**1.49E-36**	**0.381**	**0.900**	**167.200**	**0.736**	**0.527**	**0.774**	**0.884**	**8.602**	**40000.000**
ADHR.1	4	2.11E-34	0.280	0.958	290.100	0.741	0.720	0.650	0.815	8.602	40000.000
ADHR.2	4	1.65E-34	0.384	0.908	168.500	0.649	0.673	0.646	0.836	8.602	40000.000
AARR.17	4	7.13E-33	0.453	0.853	117.300	0.595	0.696	0.630	0.829	8.854	20642.857
ADHR.9	4	1.89E-24	0.403	0.910	121.100	0.587	0.722	0.705	0.873	8.854	20642.857
AADR.4	4	7.15E-26	0.431	0.866	98.200	0.603	0.701	0.650	0.832	8.268	18519.000
ADHR.3	4	3.30E-34	0.398	0.900	157.400	0.664	0.623	0.696	0.855	8.268	18519.000
ADRR.15	4	1.11E-22	0.421	0.883	94.600	0.787	0.769	0.602	0.842	7.602	4000.000
AHRR.19	4	2.96E-38	0.400	0.883	158.200	0.583	0.631	0.682	0.851	7.602	4000.000
AHRR.24	4	2.53E-37	0.318	0.933	225.300	0.390	0.660	0.636	0.814	7.770	3317.647
AAHR.5	4	7.79E-32	0.437	0.865	120.300	0.676	0.568	0.738	0.873	7.745	2450.000
AARR.15	4	6.58E-30	0.487	0.829	97.200	0.738	0.564	0.757	0.878	7.745	2450.000

ADHR.7	4	8.78E-27	0.360	0.928	154.700	0.507	0.775	0.649	0.858	7.745	2450.000
ADRR.13	4	3.03E-22	0.447	0.873	87.900	0.728	0.712	0.690	0.871	7.745	2450.000
AAHR.3	4	1.05E-34	0.400	0.887	147.200	0.614	0.663	0.643	0.813	7.959	2400.000
AADR.12	4	6.56E-19	0.486	0.852	67.700	0.597	0.702	0.669	0.855	7.328	2128.000
AADR.7	4	3.07E-27	0.414	0.883	113.200	0.653	0.751	0.625	0.807	7.328	2128.000
AARR.41	4	8.52E-29	0.497	0.830	93.700	0.705	0.683	0.648	0.823	7.328	2128.000
ADRR.12	4	1.46E-29	0.370	0.921	159.400	0.613	0.747	0.658	0.840	7.328	2128.000
ADRR.17	4	6.02E-24	0.392	0.901	111.200	0.457	0.675	0.694	0.892	7.328	2128.000
ADRR.3	4	2.53E-28	0.504	0.825	90.500	0.839	0.711	0.617	0.807	7.328	2128.000
AADH.4	4	1.08E-20	0.378	0.908	101.400	0.602	0.539	0.810	0.922	7.009	1020.000
AARR.14	4	7.09E-30	0.473	0.844	102.900	0.670	0.727	0.613	0.813	7.009	1020.000
ADHR.17	4	9.83E-37	0.256	0.959	315.300	0.576	0.635	0.695	0.842	7.009	1020.000
ADRR.6	4	3.22E-23	0.483	0.865	87.900	0.763	0.620	0.769	0.888	7.009	1020.000

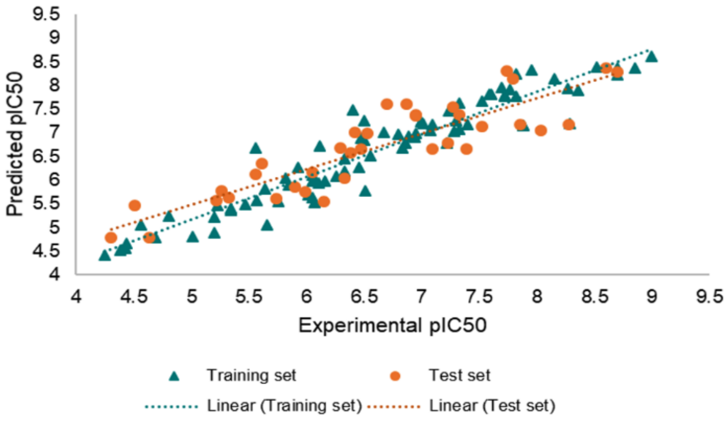

Figure S1. Plot of experimental versus predicted pIC50 values for atom based 3D QSAR Pharm-1

Table S3. The canonical smiles, activity and selectivity index for 126 MAO inhibitors

ID	Class	Canonical smiles	pIC50-MAO-B	SI	Cluster	References
1*	Selective	c1ccc(cc1)OCCOc2ccc3c(c2)C(=O)CC3	9.000	1348.000	7	56
2*	Selective	c1cc(ccc1COc2ccc3c(c2)NC(=O)CC3)Cl	8.854	20642.857	12	58
3	Selective	c1cc(cc(c1)Br)COc2ccc3c(c2)C(=O)CC3	8.699	58.000	1	56
4	Nonselective	c1cc(cc(c1)Cl)COc2ccc3c(c2)C(=O)CC3	8.699	42.000	1	56
5	Nonselective	c1cc(ccc1COc2ccc3c(c2)C(=O)CC3)Cl	8.699	16.000	1	56
6*	Selective	c1cc(ccc1COc2ccc3c(c2)NC(=O)CC3)Br	8.602	40000.000	12	58
7	Nonselective	c1cc(ccc1COc2ccc3c(c2)C(=O)CC3)Br	8.523	13.000	1	56
8	Nonselective	COc1ccc2c(c1)C/C(=C\c3ccc(o3)Br)/C2=O	8.357	41.591		51
9	Selective	Cc1ccc(cc1)/C=C/2\Cc3cc(ccc3C2=O)O	8.284	619.231	9	52
10	Selective	c1cc(ccc1/C=C/2\Cc3cc(ccc3C2=O)O)Br	8.276	198.113	9	52
11*	Selective	c1cc(ccc1COc2ccc3c(c2)CCC(=O)N3)Br	8.268	18519.000	12	58
12	Nonselective	c1cc(ccc1OCCOc2ccc3c(c2)C(=O)CC3)Cl	8.155	23.000	1	56
13*	Selective	COc1ccc2c(c1)C/C(=C\c3ccccc3)/C2=O	8.036	10870.000	9	52
14*	Selective	Cc1cccc(c1)COc2ccc3c(c2)NC(=O)CC3	7.959	2400.000	8	58
15	Selective	c1cc(ccc1/C=C/2\Cc3cc(ccc3C2=O)O)Cl	7.886	57.000		52
16	Nonselective	c1cc(cc(c1)Br)/C=C/2\Cc3cc(ccc3C2=O)O	7.886	10.077		52
17	Nonselective	c1cc(c(cc1/C=C/2\Cc3cc(ccc3C2=O)O)Cl)Cl	7.854	16.786	9	52
18	Selective	c1cc(cc(c1)Cl)COc2ccc3c(c2)CCC3=O	7.824	170.667	1	56
19	Nonselective	c1ccc(cc1)COc2ccc3c(c2)C(=O)CC3	7.824	37.000	7	56
20	Nonselective	c1cc(ccc1OCCOc2ccc3c(c2)C(=O)CC3)Br	7.796	20.188	1	56
21*	Selective	c1cc(ccc1COc2ccc3c(c2)CCC(=O)N3)Cl	7.770	3317.647	12	58
22*	Selective	Cc1ccc(cc1)COc2ccc3c(c2)NC(=O)CC3	7.745	2450.000	8	58
23	Selective	c1cc(cc(c1)Br)COc2ccc3c(c2)CCC3=O	7.721	161.474	1	56
24	Nonselective	c1ccc(cc1)CCCOc2ccc3c(c2)C(=O)CC3	7.721	10.684	7	56
25	Nonselective	CC(C)CCCOc1ccc2c(c1)C(=O)CC2	7.699	18.200	7	56
26*	Selective	Cc1ccc(cc1)CCOc2ccc3c(c2)CCC(=O)N3	7.602	4000.000	8	58
27	Selective	Cc1ccc(o1)/C=C/2\Cc3cc(ccc3C2=O)OC	7.585	181.923	9	51

28	Nonselective	c1cc2c(cc1OCCC3CCCCC3)C(=O)CC2	7.523	25.233	9	56
29	Nonselective	c1cc(cc(c1)Cl)/C=C/2\Cc3cc(ccc3C2=O)O	7.523	5.233	7	52
30	Nonselective	CN(C)c1ccc(cc1)/C=C/2\Cc3ccccc3C2=O	7.509	49.032	9	52
31	Selective	c1ccc(cc1)CCCOc2ccc3c(c2)CCC3=O	7.398	57.400	7	56
32	Selective	c1cc(cc(c1)Cl)COc2ccc3c(c2)CCC3	7.387	76.585	2	56
33*	Selective	c1ccc(cc1)OCCOc2ccc3c(c2)NC(=O)CC3	7.328	2128.000	8	58
34	Selective	Cc1ccc(cc1)CCOc2ccc3c(c2)NC(=O)CC3	7.328	734.043	9	58
35	Nonselective	c1cc(ccc1/C=C/2\Cc3cc(ccc3C2=O)O)F	7.328	14.447	8	52
36	Nonselective	c1cc2c(cc1OCC3CCCCC3)CCC2=O	7.301	19.420	7	56
37	Nonselective	Cc1cccc(c1)/C=C/2\Cc3cc(ccc3C2=O)O	7.284	16.538	9	52
38	Nonselective	c1cc(ccc1COc2ccc3c(c2)CCC3=O)Br	7.284	12.673	1	56
39	Nonselective	c1cc2c(cc1OCCC3CCCCC3)CCC2=O	7.276	30.321	7	56
40	Nonselective	c1cc(ccc1COc2ccc3c(c2)CCC3=O)Cl	7.237	8.690	1	56
41	Selective	c1cc2c(cc1OCCC3CCCCC3)CCC2	7.229	66.373		56
42	Selective	CN(C)c1ccc(cc1)/C=C/2\Cc3cc(ccc3C2=O)OC	7.222	191.667	9	52
43	Nonselective	c1cc(cc(c1)Br)COc2ccc3c(c2)CCC3	7.092	48.963	7	56
44	Nonselective	CC(C)CCCOc1ccc2c(c1)CCC2=O	7.092	6.630	2	56
45	Nonselective	c1cc(cc(c1)F)/C=C/2\Cc3cc(ccc3C2=O)O	7.076	5.452	9	52
46*	Selective	Cc1ccc(cc1)COc2ccc3c(c2)CCC(=O)N3	7.009	1020.000	8	58
47	Selective	c1ccc(cc1)OCCOc2ccc3c(c2)CCC(=O)N3	6.991	980.000	8	58
48	Nonselective	COc1ccc2c(c1)C/C(=C\c3ccccn3)/C2=O	6.951	2.696	9	51
49	Selective	c1cc(ccc1OCCOc2ccc3c(c2)NC(=O)CC3)Cl	6.947	885.000	12	58
50	Nonselective	c1ccc(cc1)OCCOc2ccc3c(c2)CCC3=O	6.947	14.009		56
51	Nonselective	COc1ccc2c(c1)C/C(=C\c3cccs3)/C2=O	6.943	45.439	9	51
52	Nonselective	c1ccc2c(c1)C/C(=C\c3ccsc3)/C2=O	6.943	13.246	6	51
53	Nonselective	c1cc(cc(c1)C#N)/C=C/2\Cc3cc(ccc3C2=O)O	6.886	3.415	9	52
54	Selective	c1ccc(cc1)COc2ccc3c(c2)CCC3=O	6.870	79.630	7	56
55	Selective	COc1ccc2c(c1)C/C(=C\C3CCCCC3)/C2=O	6.857	719.000	9	51
56	Nonselective	c1cc(ccc1COc2ccc3c(c2)CCC3)Br	6.830	22.493	2	56
57	Nonselective	c1cc(ccc1OCCOc2ccc3c(c2)CCC3=O)Br	6.796	5.375	1	56
58	Selective	Cc1cccc(c1)CCOc2ccc3c(c2)CCC(=O)N3	6.703	198.485	8	58

59	Nonselective	CC(C)c1ccc(cc1)/C=C/2\Cc3cc(ccc3C2=O)O	6.668	4.470	9	52
60	Nonselective	c1ccc2c(c1)C/C(=C\C3CCCCC3)/C2=O	6.640	22.751	6	51
61	Nonselective	COc1ccc(cc1)C(=O)c2c(c3ccccc3s2)O	6.553	14.929	3	53
62	Nonselective	c1cc(ccc1OCCOc2ccc3c(c2)CCC3=O)Cl	6.526	2.725	1	56
63	Nonselective	c1ccc(cc1)OCCOc2ccc3c(c2)CCC3	6.510	30.136		56
64	Selective	Cc1cccc(c1)COc2ccc3c(c2)CCC(=O)N3	6.504	99.042	8	58
65	Nonselective	c1cc(ccc1/C=C/2\Cc3cc(ccc3C2=O)O)C#N	6.500	6.582	9	52
66	Selective	c1ccc(cc1)COc2ccc3c(c2)CCC3	6.475	105.418		56
67	Nonselective	COc1ccc2c(c1)C/C(=C\c3cccnc3)/C2=O	6.471	25.296	9	51
68	Selective	c1ccc2c(c1)c(c(s2)C(=O)c3cccc(c3)Br)O	6.456	180.571	5	53
69	Nonselective	c1ccc(cc1)/C=C/2\Cc3cc(ccc3C2=O)O	6.425	5.160	9	52
70	Nonselective	c1ccc2c(c1)C/C(=C\c3ccc[nH]3)/C2=O	6.421	0.850	4	51
71	Nonselective	COc1ccc2c(c1)C/C(=C\c3ccco3)/C2=O	6.405	5.914	9	51
72	Nonselective	CC(C)CCCOc1ccc2c(c1)CCC2	6.384	18.373		56
73	Nonselective	c1ccc2c(c1)C/C(=C\c3cccs3)/C2=O	6.379	3.182		51
74	Nonselective	c1ccc2c(c1)C/C(=C\c3cccnc3Cl)/C2=O	6.333	1.013		51
75	Nonselective	Cc1ccc(cc1)C(=O)c2c(c3ccccc3s2)O	6.328	5.766	4	53
76	Nonselective	COc1cccc(c1)/C=C/2\Cc3cc(ccc3C2=O)O	6.327	2.527	9	52
77	Nonselective	c1cc(ccc1COc2ccc3c(c2)CCC3)Cl	6.291	7.980	2	56
78	Selective	c1ccc2c(c1)c(c(s2)C(=O)c3cccc(c3)Cl)O	6.260	92.727	5	53
79	Selective	c1cc(ccc1Cn2cnc3ccc(cc3c2=O)OCc4ccc(cc4)F)F	6.164	145.000		57
80	Nonselective	COc1ccc2c(c1)C(=O)/C(=C/c3ccccc3)/CC2	6.151	2.772	9	54
81	Nonselective	CN(C)c1ccc(cc1)/C=C/2\Cc3cc(ccc3C2=O)O	6.115	2.240	9	52
82	Nonselective	COc1cccc(c1)C(=O)c2c(c3ccccc3s2)O	6.108	42.308	3	53
83	Selective	c1cc(ccc1Cn2cnc3ccc(cc3c2=O)OCc4ccc(cc4)C#N)C#N	6.072	118.000		57
84	Nonselective	c1ccc2c(c1)c(c(s2)C(=O)c3ccc(cc3)Br)O	6.056	23.977	5	53
85	Nonselective	c1ccc2c(c1)C/C(=C\c3ccco3)/C2=O	6.051	1.350		51
86	Nonselective	c1ccc2c(c1)c(c(s2)C(=O)c3ccc(cc3)Cl)O	6.051	15.506	5	53
87	Nonselective	COc1ccc2c(c1)C(=O)/C(=C/C3CCCCC3)/CC2	6.048	5.128	9	54
88	Nonselective	c1cc(cc(c1)O)/C=C/2\Cc3cc(ccc3C2=O)O	6.015	1.242	9	52
89	Nonselective	COc1ccc2c(c1)C(=O)/C(=C/c3ccsc3)/CC2	5.987	3.748	9	54

90	Nonselective	c1ccc2cc(ccc2c1)C(=O)c3c(c4ccccc4s3)O	5.967	39.352	5	53
91	Nonselective	c1ccc(cc1)CCCOc2ccc3c(c2)CCC3	5.928	7.768		56
92	Nonselective	c1ccc2c(c1)C/C(=C\c3cccnc3)/C2=O	5.896	0.048		51
93	Nonselective	c1ccc2c(c1)c(c(s2)C(=O)c3cccc(c3)F)O	5.842	17.431	4	53
94	Nonselective	c1ccc2c(c1)C/C(=C\c3ccccn3)/C2=O	5.815	0.558		51
95	Nonselective	COc1ccc2c(c1)C(=O)/C(=C/c3cccs3)/CC2	5.757	3.446	9	54
96	Nonselective	Cc1cccc(c1)C(=O)c2c(c3ccccc3s2)O	5.742	6.961	4	53
97	Nonselective	CN1Cc2cc(ccc2NC1=O)C(=O)c3ccc(cc3)Cl	5.654	10.946		55
98	Nonselective	c1ccc2c(c1)c(c(s2)C(=O)c3ccc(cc3)F)O	5.642	5.877	4	53
99	Nonselective	c1ccc(cc1)/C=C/2\Cc3ccc(cc3C2=O)O	5.616	0.182	9	52
100	Nonselective	c1ccc(cc1)/C=C/2\Cc3ccccc3C2=O	5.590	2.113	6	52
101	Nonselective	COc1ccc2c(c1)C(=O)/C(=C/c3cccnc3)/CC2	5.570	3.056	9	54
102	Nonselective	c1cc(ccc1/C=C/2\Cc3cc(ccc3C2=O)O)O	5.562	0.609	9	52
103	Nonselective	c1ccc2c(c1)c(c(s2)C(=O)c3ccc(cc3)N(=O)O)O	5.561	6.836	3	53
104	Nonselective	COc1ccc2c(c1)C(=O)/C(=C/c3ccccn3)/CC2	5.472	2.626	9	54
105	Nonselective	c1ccc2c(c1)c(c(s2)C(=O)c3ccc(cc3)C#N)O	5.346	4.701	5	53
106	Nonselective	COc1ccc2c(c1)C(=O)/C(=C/c3ccco3)/CC2	5.344	1.393	9	54
107	Nonselective	COc1ccc2c(c1)C(=O)/C(=C/c3ccc[nH]3)/CC2	5.329	3.092	9	54
108	Nonselective	COc1ccc2c(c1)C(=O)/C(=C/C3CCCC3)/CC2	5.263	5.916	9	54
109	Nonselective	c1ccc(cc1)c2ccc(cc2)C(=O)c3c(c4ccccc4s3)O	5.253	6.530	5	53
110	Nonselective	c1cc(ccc1OCCOc2ccc3c(c2)CCC3)Br	5.228	4.339		56
111	Nonselective	COc1ccc2c(c1)C(=O)/C(=C/c3ccncc3)/CC2	5.219	0.960	9	54
112	Nonselective	c1ccc2c(c1)CC/C(=C\c3cccnc3)/C2=O	5.203	3.738	6	54
113	Nonselective	c1cc(cc(c1)I)COc2ccc3c(c2)c(=O)[nH]cn3	5.197	15.000		57
114	Nonselective	c1ccc(cc1)C(=O)c2c(c3ccccc3s2)O	5.131	1.800	4	53
115	Nonselective	c1ccc2c(c1)c(c(s2)C(=O)c3ccsc3)O	5.121	0.852	6	53
116	Nonselective	CN1Cc2cc(ccc2NC1=O)C(=O)c3cccc(c3)F	5.015	5.176	11	55
117	Nonselective	c1cc(ccc1COc2ccc3c(c2)c(=O)[nH]cn3)C#N	4.801	6.000		57
118*	Nonselective	CN1Cc2cc(ccc2NC1=O)C(=O)c3ccccc3F	4.690	3.510	11	55
119*	Nonselective	Cc1ccccc1C(=O)c2c(c3ccccc3s2)O	4.631	0.799	4	53
120*	Nonselective	CN1Cc2cc(ccc2NC1=O)C(=O)c3ccc(cc3)F	4.565	2.018	11	55

121*	Nonselective	CN1Cc2cc(ccc2NC1=O)C(=O)c3ccccc3	4.511	1.679	10	55
122*	Nonselective	CN1Cc2cc(ccc2NC1=O)C(=O)Cc3ccccc3	4.434	1.383	10	55
123*	Nonselective	c1ccc2c(c1)c(c(s2)C(=O)c3ccc(cc3Cl)Cl)O	4.428	1.164	5	53
124*	Nonselective	c1cc(cc(c1)I)Cn2cnc3ccc(cc3c2=O)OCc4cccc(c4)I	4.383	2.400		57
125*	Nonselective	c1ccc2c(c1)c(c(s2)C(=O)c3cccnc3)O	4.304	1.008	4	53
126*	Nonselective	c1ccc2c(c1)c(c(s2)C(=O)c3ccncc3)O	4.245	0.946	4	53

*compounds that participated to the pharmacophore generation; compounds without cluster integration are singletons

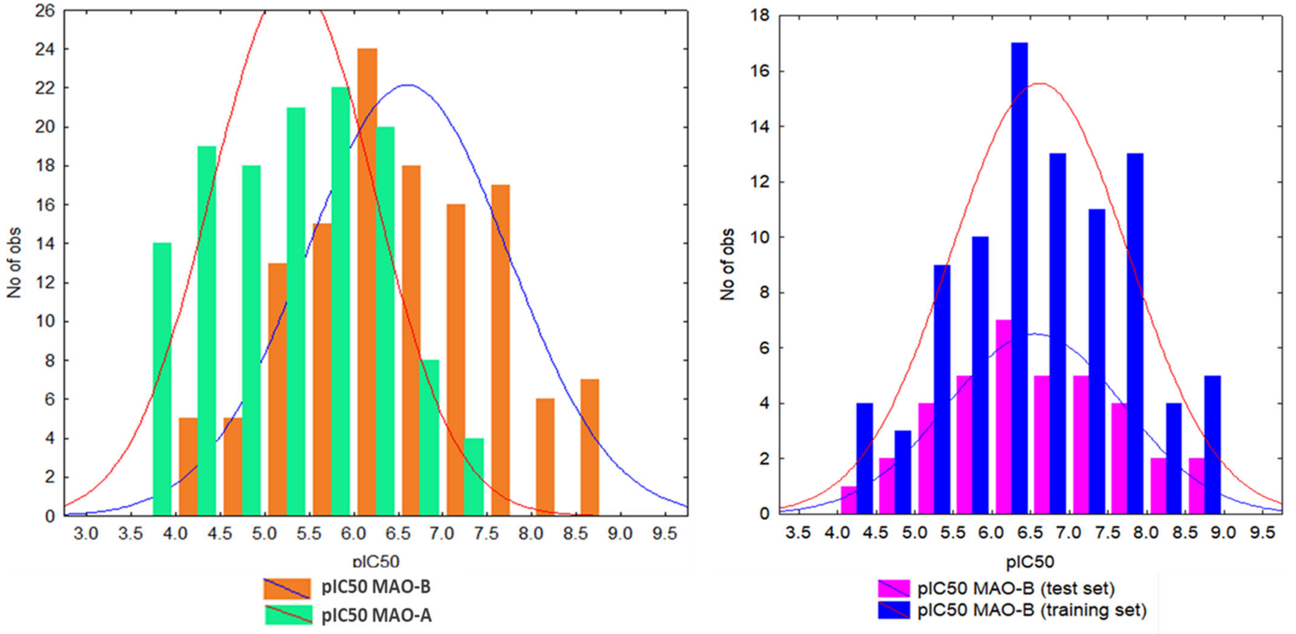

(a) (b)

Figure S2. The distribution of biological activity (pIC50) against MAO-A and MAO-B (STATISTICA 7.1, Tulsa, StatSoft Inc, OK, USA) (a); The distribution of biological activity (pIC50) against MAO-B for training and test set (STATISTICA 7.1, Tulsa, StatSoft Inc, OK, USA) (b)

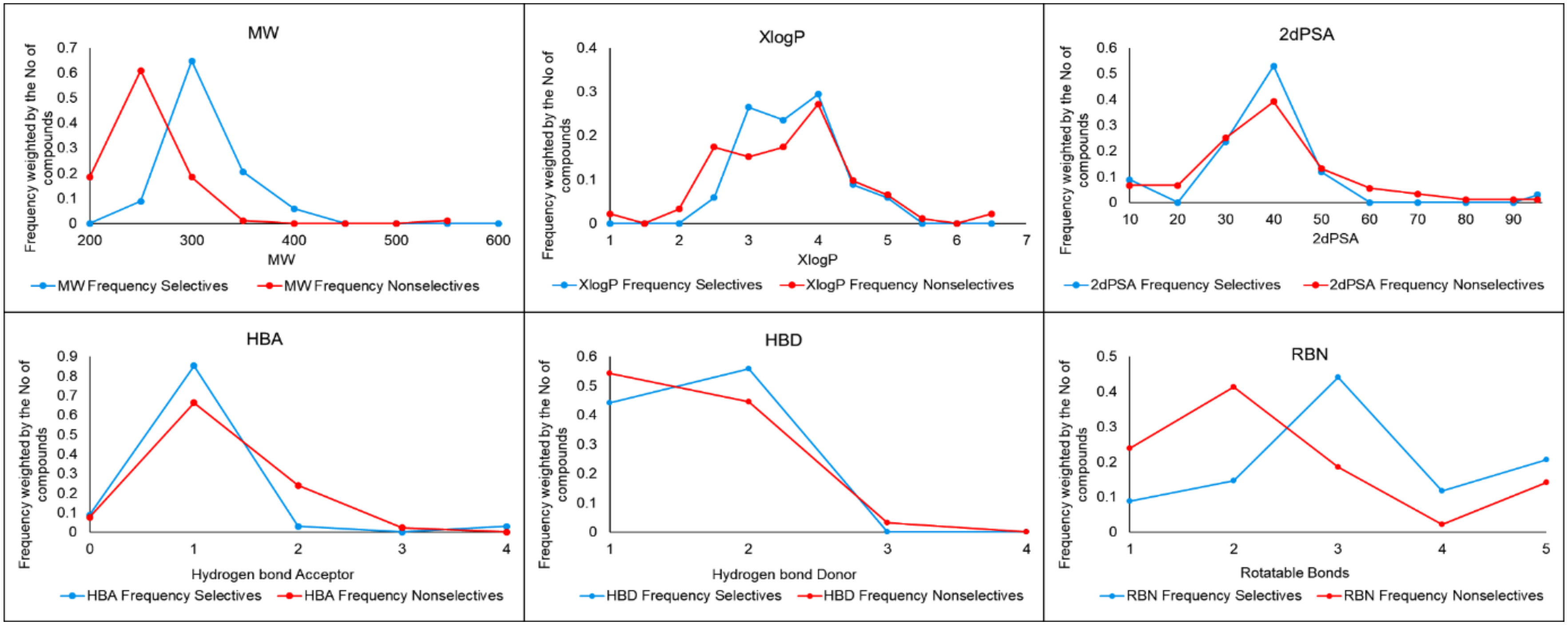

Figure S3. The distribution of drug-like properties for 126 MAO-A/B inhibitors

Table S4. The statistical parameters used for internal and external model validation.*

No	Equations used for internal validation	No	Equations used for external validation
S1	$r^2 = 1 - \frac{\sum_{i=1}^{n}(\hat{y}_i - y_i)}{\sum_{i=1}^{n}(y_i - \bar{y})^2}$	S6	$r^2_{pred} = 1 - \frac{\sum_{i=1}^{n_{pred}}(y_i - \hat{y}_{i\,pred})^2}{\sum_{i=pred}^{n_e}(y_i - \bar{y})^2}$
S2	$q^2 = 1 - \frac{\sum_{i=1}^{n}(\hat{y}_{i/i} - y_i)}{\sum_{i=1}^{n}(y_i - \bar{y})^2}$	S7	$Q^2_{F1} = 1 - \frac{\sum_{i=1}^{n_{ext}}(y_i - \hat{y}_i)^2}{\sum_{i=1}^{n_{ext}}(y_i - \bar{y}_{tr})^2}$ $Q^2_{F2} = 1 - \frac{\sum_{i=1}^{n_{ext}}(y_i - \hat{y}_i)^2}{\sum_{i=1}^{n_{ext}}(y_i - \bar{y}_{ext})^2}$

			$Q_{F3}^2 = 1 - \frac{\left[\sum_{i=1}^{n_{ext}}(y_i - \hat{y}_i)^2\right] / n_{ext}}{\left[\sum_{i=1}^{n_{tr}}(y_i - \bar{y}_{TR})^2\right] / n_{tr}}$
S3	$RMSE_{tr} = \sqrt{\frac{\sum_{i=1}^{n_{tr}}(y_i - \hat{y}_i)^2}{n_{tr}}}$	S8	$RMSE_{pred} = \sqrt{\frac{\sum_{i=1}^{n_{pred}}(y_i - \hat{y}_i)^2}{n_{pred}}}$
S4	$MAE_{tr} = \frac{\sum_{i=1}^{n_{tr}} \lvert y_i - \hat{y}_i \rvert}{n_{tr}}$	S9	$MAE_{pred} = \frac{\sum_{i=1}^{n_{pred}} \lvert y_i - \hat{y}_i \rvert}{n_{pred}}$
S5	$CCC_{tr} = \frac{2\sum_{i=1}^{n_{tr}}(y_i - \bar{y})(\hat{y}_i - \bar{\hat{y}})}{\sum_{i=1}^{n_{tr}}(y_i - \bar{y})^2 + \sum_{i=1}^{n_{tr}}(\hat{y}_i - \bar{\hat{y}})^2 + n_{tr}(\bar{y} - \bar{\hat{y}})^2}$	S10	$CCC_{pred} = \frac{2\sum_{i=1}^{n_{pred}}(y_i - \underline{y})(\hat{y}_i - \underline{\hat{y}})}{\sum_{i=1}^{n_{pred}}(y_i - \underline{y})^2 + \sum_{i=1}^{n_{pred}}(\hat{y}_i - \underline{\hat{y}})^2 + n_{pred}(\underline{y} - \underline{\hat{y}})^2}$

*r^2- Squared Correlation Coefficient for fitting; r_{pred}^2 - Squared Correlation Coefficient for prediction set; q - Leave-n-Out Correlation Coefficient; Q_{F1}^2 , Q_{F2}^2, Q_{F3}^2 - similar to q values; $RMSE_{tr}$ and $RMSE_{pred}$ - Root-Mean-Square Errors for the training set, and for the prediction set, respectively; MAE_{tr} and MAE_{pred} - Mean Absolute Error for the training set and prediction set, respectively; CCC_{tr} and CCC_{pred} - Concordance Correlation Coefficient for the training set and for the prediction set, respectively; y_i represents is the observed response variable; $\underline{y}$ is its average; $\hat{y}_i$ expresses the corresponding predicted value; $\hat{y}_{i/i}$ is the predicted value of the response calculated excluding the ith element from the model (the leave-n-out technique); $\underline{\hat{y}}$ is the average of the predicted data values. In all equations, *tr* refers to the training set and *pred* refers to the prediction set.

TableS5. Fingerprints with ROC analyses of the compounds (cpds) of the dataset

		**Category A**		
BitVector_623	BitVector_3	BitVector_642	BitVector_949	BitVector_290
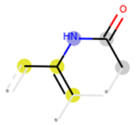	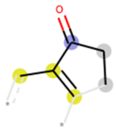	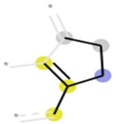	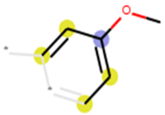	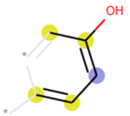
AUC=0.962/14cpds	AUC=0.683/27cpds	AUC=0.675/48cpds	AUC=0.444/21cpds	AUC=0.351/18cpds
		**Category B**		
BitVector_681		BitVector_562		BitVector_453
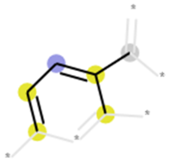		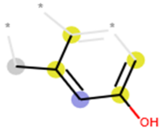		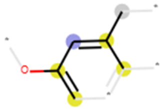
AUC=0.567/38cpds		AUC=0.492/33cpds		AUC=0.447/77cpds
		**Category C**		

BitVector_478	BitVector_677	BitVector_609	BitVector_410
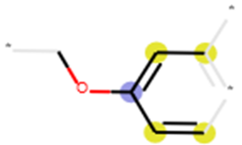	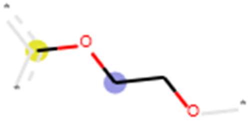	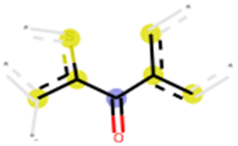	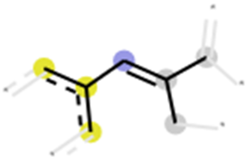
AUC=0.811/52cpds	AUC=0.603/14cpds	AUC=0.425/17cpds	AUC=0.383/30cpds
**Category D**			
BitVector_910	BitVector_217	BitVector_681	BitVector_961
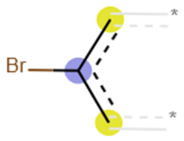	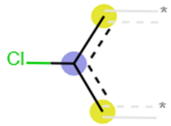	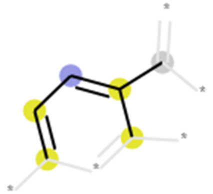	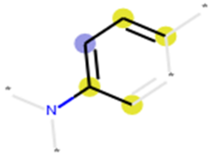
AUC=0.664/15cpds	AUC=0.645/17cpds	AUC=0.567/38cpds	AUC=0.564/7cpds

* the “cpds” abbreviation is associated with,"compounds"

Table S6. The interactions of selective (**2, 6, 13**) and nonselective (**92, 99, 111**) MAOB inhibitors with MAOB binding site residues.

**Aminoacid**	**Distance (Å)**	**Category**	**Type**
Selective MAOB inhibitors			
Compound **2**			
TYR435	1.447	Hydrogen Bond	Conventional Hydrogen Bond
ILE199	2.525	Hydrogen Bond	Carbon Hydrogen Bond
TYR326	2.900	Hydrogen Bond	Carbon Hydrogen Bond
PRO104	3.287	Hydrogen Bond	Carbon Hydrogen Bond
GLN206	3.519	Hydrogen Bond	Pi-Donor Hydrogen Bond
TYR326	2.931	Hydrophobic	Pi-Sigma
LEU171	3.970	Hydrophobic	Pi-Sigma
CYS172	5.492	Other	Pi-Sulfur
PRO104	4.037	Hydrophobic	Alkyl
LEU164	3.873	Hydrophobic	Alkyl

ILE316	4.697	Hydrophobic	Alkyl
ILE199	4.020	Hydrophobic	Pi-Alkyl
ILE316	4.642	Hydrophobic	Pi-Alkyl
TRP119	5.311	Hydrophobic	Pi-Alkyl
Compound **6**			
TYR435	1.521	Hydrogen Bond	Conventional Hydrogen Bond
ILE199	2.597	Hydrogen Bond	Carbon Hydrogen Bond
TYR326	2.813	Hydrogen Bond	Carbon Hydrogen Bond
PRO104	3.332	Hydrogen Bond	Carbon Hydrogen Bond
GLN206	3.524	Hydrogen Bond	Pi-Donor Hydrogen Bond
CYS172	5.530	Other	Pi-Sulfur
PRO104	4.087	Hydrophobic	Alkyl
LEU164	3.879	Hydrophobic	Alkyl
ILE316	4.724	Hydrophobic	Alkyl
LEU171	4.174	Hydrophobic	Pi-Alkyl
ILE199	3.956	Hydrophobic	Pi-Alkyl
ILE316	4.742	Hydrophobic	Pi-Alkyl
TRP119	5.272	Hydrophobic	Pi-Alkyl
Compound **13**			
CYS172	3.188	Hydrogen Bond	Conventional Hydrogen Bond
ILE199	3.940	Hydrophobic	Pi-Sigma
ILE199	3.867	Hydrophobic	Pi-Sigma
ILE316	3.721	Hydrophobic	Pi-Sigma
TYR326	2.554	Hydrophobic	Pi-Sigma
CYS172	5.960	Other	Pi-Sulfur
ILEU171	4.220	Hydrophobic	Pi-Alkyl
Nonselective MAO-B inhibitors			
Compound **92**			
CYS172	3.438	Hydrogen Bond	Conventional Hydrogen Bond

ILE199	2.808	Hydrogen Bond	Carbon Hydrogen Bond
TYR326	2.467	Hydrogen Bond	Carbon Hydrogen Bond
TYR398	5.707	Hydrophobic	Pi-Pi Stacked
LEU171	5.029	Hydrophobic	Pi-Alkyl
LEU171	5.399	Hydrophobic	Pi-Alkyl
ILE199	4.055	Hydrophobic	Pi-Alkyl
ILE316	5.143	Hydrophobic	Pi-Alkyl
Compound **99**			
CYS172	4.955	Other	Pi-Sulfur
TYR398	5.671	Hydrophobic	Pi-Pi Stacked
ILE199	3.772	Hydrophobic	Pi-Alkyl
ILE316	5.189	Hydrophobic	Pi-Alkyl
LEU171	5.165	Hydrophobic	Pi-Alkyl
ILE198	5.487	Hydrophobic	Pi-Alkyl
Compound **111**			
CYS172	3.326	Hydrogen Bond	Conventional Hydrogen Bond
PRO102	2.872	Hydrogen Bond	Carbon Hydrogen Bond
CYS172	2.42	Hydrogen Bond	Carbon Hydrogen Bond
ILE199	3.941	Hydrophobic	Pi-Sigma
CYS172	5.933	Other	Pi-Sulfur
TYR398	5.914	Hydrophobic	Pi-Pi Stacked
LEU171	4.437	Hydrophobic	Alkyl
LEU171	4.821	Hydrophobic	Pi-Alkyl
ILE316	4.546	Hydrophobic	Pi-Alkyl
TYR326	3.504	Hydrophobic	Pi-Alkyl
